# Chatbot-Delivered Real-Time Support to Improve HIV Self-Testing Rates: A Randomized Clinical Trial

**DOI:** 10.1001/jamanetworkopen.2025.44821

**Published:** 2025-11-24

**Authors:** Siyu Chen, Fuk-yuen Yu, Yuan Fang, Jerome Yau, Neda Ng, Qingpeng Zhang, Zhao Ni, Minh Cuong Duong, Fenghua Sun, Phoenix K. H. Mo, Zixin Wang

**Affiliations:** 1 Centre for Health Behaviours Research, JC School of Public Health and Primary Care, Chinese University of Hong Kong, Hong Kong SAR, China; 2 Department of Health and Physical Education, Education University of Hong Kong, Hong Kong SAR, China; 3 AIDS Concern, Hong Kong SAR, China; 4 Musketeers Foundation Institute of Data Science, University of Hong Kong, Hong Kong SAR, China; 5 Department of Pharmacology and Pharmacy, LKS Faculty of Medicine, University of Hong Kong, Hong Kong SAR, China; 6 School of Nursing, Yale University, New Haven, Connecticut; 7 Centre for Interdisciplinary Research on AIDS (CIRA), Yale University, New Haven, Connecticut; 8 School of Population Health, University of New South Wales, Sydney, New South Wales, Australia

## Abstract

**Importance:**

HIV self-testing (HIVST) with online real-time support by human administrators (HIVST-OIC) is an evidence-based HIV intervention for men who have sex with men (MSM) but requires intensive resources to implement.

**Objective:**

To investigate whether a novel HIVST service using a chatbot to deliver online real-time instruction and counseling support (HIVST-chatbot) is noninferior to the HIVST-OIC in increasing HIVST uptake and counseling support for MSM.

**Design, Setting, and Participants:**

A partially blinded, parallel-group, and noninferiority randomized clinical trial was conducted between April 16, 2023, and May 31, 2024, in Hong Kong, China. Eligible participants were Hong Kong–based, Chinese-speaking MSM 18 years or older who reported anal intercourse with men in the past 6 months and had access to WhatsApp. Participants were recruited via gay venues, online recruitment, and peer referrals and then randomized to the HIVST-chatbot or HIVST-OIC groups. Both intention-to-treat (ITT) and complete case analyses were performed. The noninferiority margin was set at 10 percentage points.

**Interventions:**

Standard-of-care pretest and posttest counseling was delivered by a chatbot for the HIVST-chatbot group and by trained human administrators in the HIVST-OIC group. Both groups received free HIVST kits.

**Main Outcomes and Measures:**

Primary outcomes included self-reported HIVST uptake and receiving any counseling support along with HIVST, validated by the research team.

**Results:**

A total of 531 participants (mean [SD] age, 34.8 [9.3] years) were randomized to either the HIVST-chatbot (n = 266) or HIVST-OIC (n = 265) groups. In ITT analysis, the HIVST-chatbot was noninferior to the HIVST-OIC in increasing HIVST uptake (216 [81.2%] vs 227 [85.7%]; proportion difference, −4.5 percentage points; 95% CI, −9.8 to 0.8 percentage points; 1-sided *P* = .10) and proportion of HIVST users who received counseling support (197 [91.2%] vs 142 [62.6%]; proportion difference, 28.7 percentage points; 95% CI, 22.5 to 34.8 percentage points; 1-sided *P* < .001) at month 6. The HIVST-chatbot was more cost-effective than HIVST-OIC.

**Conclusions and Relevance:**

In this randomized clinical trial, the HIVST-chatbot was noninferior to the HIVST-OIC in increasing HIVST uptake and counseling among HIVST users. These results suggest that the HIVST-chatbot might be an alternative to the evidence-based HIVST-OIC service for MSM.

**Trial Registration:**

ClinicalTrial.gov Identifier: NCT05796622

## Key Points

**Question:**

Is an HIV self-testing (HIVST) service with chatbot-delivered real-time counseling (HIVST-chatbot) noninferior to an evidence-based HIVST online service with human administrator–delivered real-time counseling (HIVST-OIC) in improving HIVST uptake and user support?

**Findings:**

In a noninferiority randomized clinical trial of 531 men who have sex with men in Hong Kong, HIVST-chatbot was noninferior to HIVST-OIC in increasing HIVST uptake and proportion of HIVST users receiving counseling support.

**Meaning:**

This study’s results suggest that HIVST-chatbot is a potentially cost-saving and scalable HIVST service for men who have sex with men.

## Teaser

This randomized clinical trial of men who have sex with men investigates whether a chatbot-delivered HIV self-testing service is similar to a self-testing service with online real-time support by human administrators in increasing self-testing uptake and counseling support.

## Introduction

HIV testing is a key strategy to control HIV.^1^ Sexually active men who have sex with men (MSM) are recommended to receive HIV testing every 3 to 6 months.^1,2^ However, the HIV testing rate among MSM in Hong Kong remains low (approximately 50% in the past 6 months).^3^ The World Health Organization (WHO) strongly recommends HIV self-testing (HIVST) as a means to increase HIV testing coverage among MSM.^4,5,6^ Many HIVST users need support. Providing free HIVST kits and user-supporting services are effective strategies to improve HIVST uptake among MSM.^5,7,8^ Both the WHO and Hong Kong Department of Health advocate the provision of sufficient support services that are adapted to local context and community preference for HIVST users.^9,10,11^

On the basis of the delivery models, support tools for HIVST services can be further categorized into active support (proactively providing counseling to all HIVST users unless they refused) or passive support (reporting results via online platforms to obtain optional posttest counseling).^12,13^ A meta-analysis^12^ showed that more than 90% of users with reactive results in programs with active support were linked to laboratory confirmation and antiretroviral therapy initiation, which was higher than those receiving passive counseling support (78.7%-79.1%). Meta-regression showed that counseling support for HIVST that involved a higher number of WHO-recommended essential components would lead to a better linkage to care.^12^

An HIVST service with online real-time instruction and standard-of-care pretest and posttest counseling provided by experienced administrators through live-chat applications (HIVST-OIC) is an evidence-based intervention and best practice for HIV prevention.^8^ A randomized clinical trial (RCT) showed that compared with promoting HIV testing in general, promoting and implementing HIVST-OIC could significantly increase HIVST uptake (87.9% vs 2.3%) and proportion of HIVST users receiving counseling support (100% vs 0%).^8^ The presence of counseling support is also helpful in reducing sexual risk behaviors among HIVST users.^14^ In the pretest counseling, the administrators will assess users’ risk of infection, inform users about their risk level, and explain the rationale. For those at high risk of infection, advice on maintaining safe sex practice will be provided. In the posttest counseling, the administrators explain the window period, provide advice for safe sex practice, and emphasize the needs of regular testing. Although the HIVST-OIC is a preferable service model among Hong Kong MSM, it requires intensive resources to implement.^8,15^

A chatbot is a computer program that replicates human interaction through various forms of communication.^16^ Chatbots could provide personalized, engaging, and on-demand health communication and are feasible and effective in promoting health behaviors.^17,18,19,20^ Surveys have found that chatbots are acceptable among MSM for promoting HIV testing.^21,22,23^ However, no study has applied chatbots to deliver real-time support for HIVST users or promote HIVST uptake.

We developed an innovative HIVST service using a chatbot to deliver online real-time instruction and counseling support (HIVST-chatbot). The primary objective of this study was to investigate whether the HIVST-chatbot would produce effects similar to the HIVST-OIC in increasing HIVST uptake and the proportion of HIVST users receiving counseling alongside testing within a 6-month period. The secondary objectives included assessing the between-group differences in the prevalence of condomless anal intercourse (CAI), multiple male sex partnerships, and uptake of other HIV testing and evaluating the cost-effectiveness of the HIVST-chatbot compared with the HIVST-OIC. We hypothesized that the HIVST-chatbot was noninferior to the HIVST-OIC in increasing both HIVST uptake and the proportion of HIVST users receiving counseling alongside testing.

## Methods

### Study Design and Participants

A partially blinded, parallel-group, noninferiority RCT was conducted between April 16, 2023, and May 31, 2024, in Hong Kong. The outcome assessors and data analysts were blinded to the participant allocation. The trial protocol and statistical plan were published before the data analysis and are available in Supplement 1.^24^ The Survey and Behavioral Research Ethics Committee of the Chinese University of Hong Kong approved the study. The RCT followed the Consolidated Standards of Reporting Trials (CONSORT) reporting guidelines.

Participants were Hong Kong–based, Chinese-speaking men 18 years or older who had anal intercourse with a man in the past 6 months and with access to WhatsApp. HIV-positive MSM were excluded. WhatsApp is the most commonly used instant messaging app in Hong Kong (78% of residents used it regularly in 2023).^25^ A noninferiority margin of 10% was defined based on consensus of a panel of stakeholders, including government officials from the Centre for Health Protection, leaders of community-based organizations (CBOs) providing HIV testing services, and researchers working in HIV prevention. Details of the procedures to define the noninferiority margin and sample size calculation are provided in eAppendix 1 in Supplement 2.

Participants were recruited through multiple sources, including outreaching in gay venues identified by the Hong Kong government (12 bars and 16 saunas),^26^ online recruitment on gay websites, and peer referral.^8,27^ Participants were guaranteed that they had the right to discontinue participation in the study at any time, that their data would be safe, and that refusal or withdrawal from the study would have no consequences. Verbal informed consent was obtained, with fieldworkers signing a pledge that participants had been fully informed about the study.^28,29^ A supermarket coupon worth HK$50 (US$6.38) was mailed to participants after completing telephone surveys at baseline (T0) and month 6 (T1).

### Randomization, Masking, and Development of the Chatbot

At the end of the baseline survey, participants were randomly assigned (1:1) into the HIVST-chatbot or the HIVST-OIC group using block randomization (block size of 8). The following considerations were taken into account when selecting such block size: (1) the block size should be a multiple of the number of groups in this RCT, (2) the target sample size (n = 528) must be divisible by the chosen block size to maintain balance within blocks, and (3) compared with smaller block size (eg, a size of 2 or 4), a larger block size could reduce the predictability of allocation sequences. Computer-generated random allocation codes were produced and sealed in opaque envelopes by a research staff without involvement in recruitment or baseline survey. One envelope was drawn and opened by the fieldworkers, who then informed the participants about their assigned group.

We used a cocreation approach to develop the chatbot system (eAppendix 2 in Supplement 2). The HIVST-chatbot is a natural language processing–based chatbot. It is not publicly available at this stage; only the HIVST-chatbot group had access to it during the project period. We integrated the chatbot with the WhatsApp platform via its public Web API (eFigure in Supplement 2). Details of the chatbot are provided in eAppendix 2 in Supplement 2.

### The HIVST-OIC and HIVST-Chatbot Groups

Participants in the HIVST-OIC group first watched an online video promoting HIV testing in general and HIVST-OIC. In the first part, a local man who identified with the MSM group introduced the benefits of HIV testing and the WHO HIV testing recommendation. In the second part, the same man narratively discussed the benefits of HIVST-OIC, demonstrated its procedures, and emphasized its real-time support for users. Participants could sign up for HIVST-OIC by informing our project staff through WhatsApp about their preference for HIVST kits (oral fluid based or blood based), service model (a comprehensive version or a simplified version), and ways to receive a free HIVST kit (eg, through rapid courier services or pick up at collaborative CBO or the research office). Participants could make an appointment with a trained HIV testing administrator to implement HIVST-OIC after receiving the kit.^8,30^ Through Line, WhatsApp, or Skype, an experienced HIV testing administrator guided the participants to perform HIVST on screen and in real time. For the comprehensive version, the administrator provided standard-of-care pretest and posttest counseling, which was identical to standard-of-care HIV testing and counseling services in Hong Kong, and explained how to use the HIVST kits. An online video demonstrating the procedures of using a HIVST kit was also available. For the simplified version, the pretest counseling was replaced by providing online pretest information, and users had access to the same demonstration videos and posttest counseling. The testing administrators would provide immediate psychological support and explain the needs to receive free confirmatory HIV testing at the Department of Health to users who received reactive results. Project staff would accompany them to visit the collaborative CBO or the Department of Health if needed.

**Figure 1.**
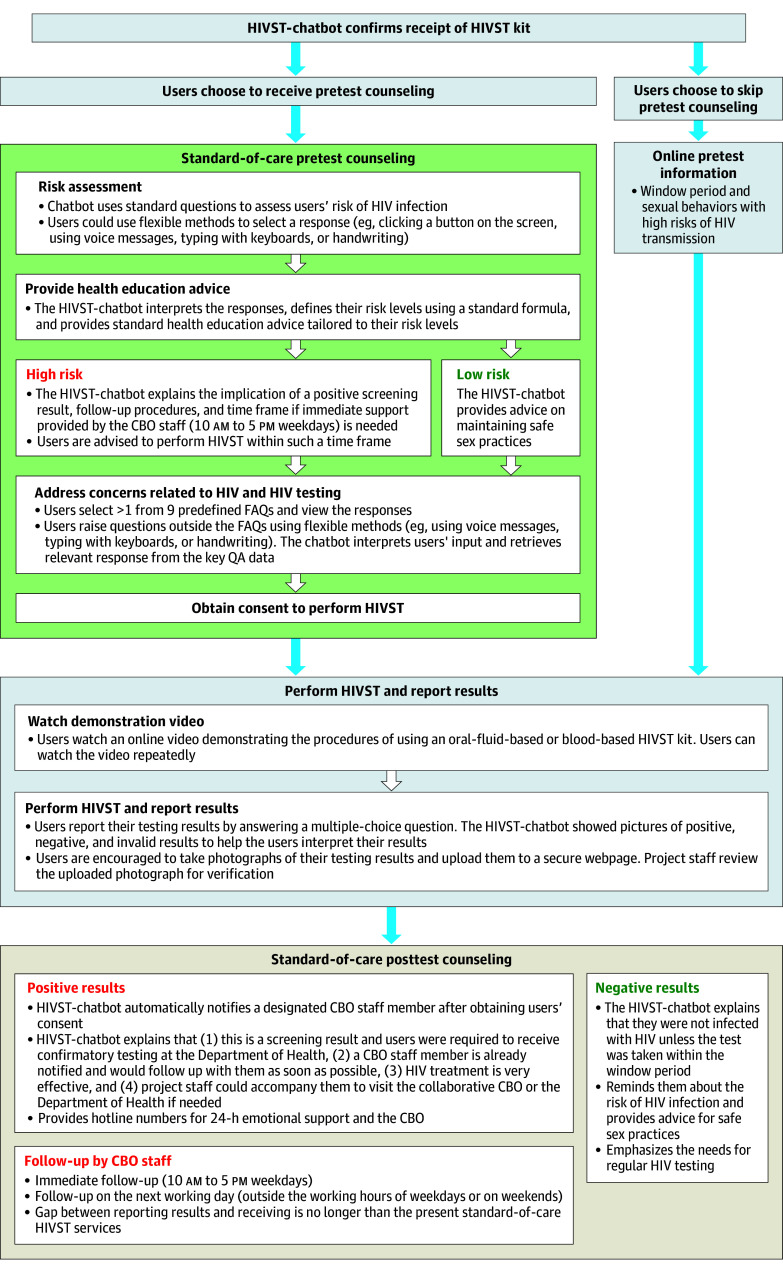
Intervention Workflow of the HIV Self-Testing Chatbot (HIVST-Chatbot) This figure illustrates the operational workflow of the HIVST-chatbot intervention, an HIVST service using a chatbot to deliver online real-time instruction and counseling support. The workflow includes pretest counseling, guidance on HIVST procedures and real-time support during test administration. Afterward, the chatbot delivers posttest counseling tailored to the user’s reported result, offers psychological support as needed, and facilitates linkage to confirmatory testing and care. CBO indicates community-based organization; FAQ, frequently asked question; QA, question-answer.

Participants in the HIVST-chatbot group first watched an online video. The first part was the same as the video watched by the HIVST-OIC group. In the second part, a local man who identified with the MSM group introduced the advantages of HIVST-chatbot (eg, 24-7 availability), demonstrated its procedures, and emphasized that real-time support would be provided by the HIVST-chatbot and CBO staff. Participants signed up for the HIVST-chatbot and received HIVST kits in the same manner as those in the HIVST-OIC group. Project staff then helped the participants connect to the chatbot. The HIVST-chatbot proactively contacted the participants to confirm the receipt of the kits. Participants could get the chatbot to implement HIVST at any time without a prior appointment. The HIVST-chatbot provided standard-of-care pretest and posttest counseling, which covered key components listed in the Quality Assurance Guideline of HIV Testing and Counseling Services, in the format of text messages and/or videos (detailed workflow shown in Figure 1).^30^ Users could skip the pretest counseling and receive the same online pretest information as those for the HIVST-OIC group.

### Study Outcomes

The primary outcomes included self-reported HIVST uptake and receiving any counseling support along with HIVST during the follow-up period. Details of the HIVST-chatbot and HIVST-OIC use were retrieved from the chat history stored in the chatbot server and records kept by the testing administrator, respectively. Secondary outcomes were CAI with men, multiple male sexual partners, and uptake of other types of HIV testing in the past 6 months measured at both T0 and T1 and cost-effectiveness of the HIVST-chatbot compared with the HIVST-OIC. In addition, levels of behavioral, cognitive, and affective engagement with the HIVST-chatbot and the HIVST-OIC among the users were measured by the Twente Engagement with eHealth Technologies Scale (TWEETS) at T1.^31^

### Statistical Analysis

There were no missing data for participants who had completed the surveys at T0 and T1. The proportion of missing data at T1 was equal to the dropout rate. Either χ^2^ tests or independent-sample *t* tests were conducted to identify between-group differences in baseline characteristics. Both complete case and intention-to-treat (ITT) analyses were performed to compare the between-group difference in primary and secondary outcomes. In the ITT analysis, assuming that the data were missing at random, a Markov Chain Monte Carlo method was used to impute missing values of the primary and secondary outcomes at T1 separately by each group.^32^ Relative risks, absolute risk reductions, numbers needed to treat, and their corresponding 95% CIs were calculated. Noninferiority was determined if the lower bound of the 1-sided 95% CI for the proportion difference in primary outcomes between the HIVST-chatbot and HIVST-OIC groups was higher than −10 percentage points. Within-group changes in outcomes (T1 vs T0) were compared using McNemar tests. Levels of engagement with HIVST-chatbot and HIVST-OIC were compared using χ^2^ tests or independent-sample *t* tests. A 2-sided *P* < .05 was considered statistically significant.

We conducted a cost-effectiveness analysis from the testing service provider’s perspective during the 6-month trial horizon. Costs were estimated using a microcosting approach, with variable costs (eg, packaging, HIVST kits, delivery, laboratory confirmation, incentives, and staff time for counseling or coordination) and fixed costs (eg, computers, desks, chairs, internet, and utilities). Fixed assets were annualized over their assumed useful life (computers: 7 years; desks and chairs: 5 years; chatbot development and maintenance: 5 years) at a 3% discount rate and prorated to the 6-month study period.^33,34^ Counseling staff costs were included for the HIVST-OIC group but not for the HIVST-chatbot group, where counseling was automated. Costs were collected in Hong Kong dollars, converted to US dollars at the 2023 average exchange rate, and reported in 2023 US dollars. Incremental cost-effectiveness ratios (ICERs) were calculated as the additional cost of the HIVST-chatbot compared with the HIVST-OIC divided by the additional number of participants achieving each primary outcome (HIVST uptake and counseling support alongside testing).^33,35^ Hong Kong’s gross domestic product per capita (US$50 500 in 2023) is conventionally used as a threshold per quality-adjusted life-year gained,^36^ and our ICERs are expressed per additional HIVST user or per HIVST user counseled. Analyses were conducted using Excel software, version 2407 (Microsoft Corp), SPSS, version 26.0 (SPSS Inc), and R, version 4.2.2 (R Foundation for Statistical Computing) from August 2024 to December 2024.

## Results

**Figure 2.**
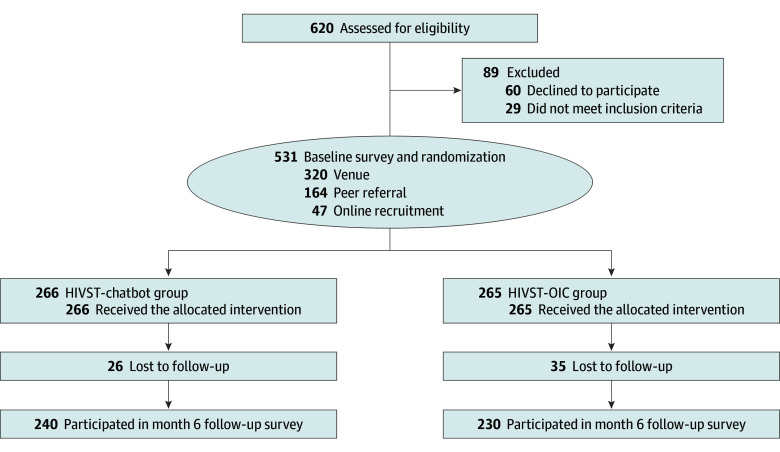
CONSORT Flow Diagram of Participants in the Trial Overview of the study design, including participant recruitment, eligibility screening, group allocation, intervention delivery, and follow-up. Eligible men who have sex with men were randomized in a 1:1 ratio into either the HIV self-testing service using a chatbot to deliver online real-time instruction and counseling support (HIVST-chatbot) group or the HIV self-testing with online real-time support by human administrators (HIVST-OIC) group. All participants completed the assessments at baseline and time 1 (month 6). The diagram presents enrollment numbers, randomization, allocation, follow-up status, and final sample sizes included in the intention-to-treat analysis across 2 groups.

A total of 620 MSM were invited to participate in the RCT; 591 met the eligibility criteria, and 531 completed the baseline survey and were randomly assigned to the HIVST-chatbot (n = 266) or HIVST-OIC groups (n = 265) (Figure 2). The mean (SD) age of the 531 participants was 34.8 (9.3) years. Baseline characteristics of the participants are presented in Table 1. The dropout rate at T1 was 9.8% in the HIVST-chatbot group and 13.2% in the HIVST-OIC group. Comparisons of baseline characteristics between dropouts and nondropouts are provided in eTable 1 in Supplement 2.

**Table 1.** Baseline Characteristics of the Study Participants

Characteristic	No. (%) of participants		
	Total (N = 531)	HIVST-chatbot (n = 266)	HIVST-OIC (n = 265)
Sociodemographic characteristics			
Age group, y			
18-24	62 (11.7)	27 (10.2)	35 (13.2)
25-34	240 (45.2)	117 (44.0)	123 (46.4)
35-44	145 (27.3)	77 (28.9)	68 (25.7)
≥45	84 (15.8)	45 (16.9)	39 (14.7)
Relationship status			
Single	435 (81.9)	216 (81.2)	219 (82.6)
Cohabited with or married to man	92 (17.3)	47 (17.7)	45 (17.0)
Cohabited with or married to woman	4 (0.8)	3 (1.1)	1 (0.4)
Educational level			
Secondary or below	85 (16.0)	45 (16.9)	40 (15.1)
Tertiary and above	446 (84.0)	221 (83.1)	225 (84.9)
Employment status			
Full time	412 (77.6)	206 (77.4)	206 (77.7)
Other	119 (22.4)	60 (22.6)	59 (22.3)
Monthly income, HK$ (US$)			
<20 000 (2564)	162 (30.5)	80 (30.1)	82 (30.9)
≥20 000 (2564)	367 (69.1)	185 (69.5)	182 (68.7)
Refuse to specify	2 (0.4)	1 (0.4)	1 (0.4)
Sexual orientation			
Gay	478 (90.0)	239 (89.8)	239 (90.2)
Bisexual	49 (9.2)	26 (9.8)	23 (8.7)
Heterosexual	2 (0.4)	1 (0.4)	1 (0.4)
Uncertain	2 (0.4)	0 (0.0)	2 (0.7)
Health conditions			
History of COVID-19			
No	99 (18.6)	52 (19.5)	47 (17.7)
Yes	432 (81.4)	214 (80.5)	218 (82.3)
COVID-19 vaccination doses			
0-1	25 (4.7)	15 (5.6)	10 (3.8)
2	71 (13.4)	28 (10.5)	43 (16.2)
≥3	435 (81.9)	223 (83.9)	212 (80.0)
STI history			
No	392 (73.8)	195 (73.3)	197 (74.3)
Yes	139 (26.2)	71 (26.7)	68 (25.7)
HIV testing in the past 6 mo			
HIV testing			
No	282 (53.1)	140 (52.6)	142 (53.6)
Yes	249 (46.9)	126 (47.4)	123 (46.4)
Use of specific type of HIV testing, yes			
HIV testing at CBOs in Hong Kong	154 (29.0)	77 (28.9)	77 (29.1)
HIV testing at governmental clinics in Hong Kong	46 (8.7)	18 (6.8)	28 (10.6)
HIV testing at private clinics in Hong Kong	7 (1.3)	5 (1.9)	2 (0.8)
HIV testing at other organizations in Hong Kong	2 (0.4)	0 (0.0)	2 (0.8)
HIV testing in places other than Hong Kong	8 (1.5)	3 (1.1)	5 (1.9)
HIV self-testing	83 (15.6)	46 (17.3)	37 (14.0)
Sexual behaviors in the past 6 mo			
Anal intercourse with regular male partner			
No	136 (25.6)	72 (27.1)	64 (24.2)
Yes	395 (74.4)	194 (72.9)	201 (75.8)
Anal intercourse with nonregular male partner			
No	287 (54.0)	145 (54.5)	142 (53.6)
Yes	244 (46.0)	121 (45.5)	123 (46.4)
Anal intercourse with male sex worker			
No	512 (96.4)	259 (97.4)	253 (95.5)
Yes	19 (3.6)	7 (2.6)	12 (4.5)
Condomless anal intercourse with men			
No	234 (44.1)	124 (46.6)	110 (41.5)
Yes	297 (55.9)	142 (53.4)	155 (58.5)
Multiple male sex partners			
No	264 (49.7)	136 (51.1)	128 (48.3)
Yes	267 (50.3)	130 (48.9)	137 (51.7)
Sexualized drug use			
No	485 (91.3)	243 (91.4)	242 (91.3)
Yes	46 (8.7)	23 (8.6)	23 (8.7)
Other HIV or STI prevention service use in the past 6 mo			
STI testing			
No	327 (61.6)	166 (62.4)	161 (60.8)
Yes	204 (38.4)	100 (37.6)	104 (39.2)
PrEP use			
No	457 (86.1)	232 (87.2)	225 (84.9)
Yes	74 (13.9)	34 (12.8)	40 (15.1)
Other HIV or STI prevention services			
No	309 (58.2)	158 (59.4)	151 (57.0)
Yes	222 (41.8)	108 (40.6)	114 (43.0)

Abbreviations: CBOs, community-based organizations; HIVST-chatbot, HIV self-testing service using a chatbot to deliver online real-time instruction and counseling support; HIVST-OIC, HIV self-testing with online real-time support by human administrators; PrEP, preexposure prophylaxis; STI, sexually transmitted infections.

In the ITT analysis, the HIVST-chatbot was noninferior to the HIVST-OIC in increasing HIVST uptake (216 [81.2%] vs 227 [85.7%]; proportion difference, −4.5 percentage points; 95% CI, −9.8 to 0.8 percentage points; 1-sided *P* = .10) and the proportion of HIVST users who received counseling support (197 [91.2%] vs 142 [62.6%]; proportion difference, 28.7 percentage points; 95% CI, 22.5 to 34.8 percentage points; 1-sided *P* < .001) at T1. More HIVST users in the HIVST-chatbot group received comprehensive counseling support than those in the HIVST-OIC group (73 [33.8%] vs 15 [6.6%]), whereas a similar proportion of HIVST users used the simplified counseling. The complete case analysis yielded similar results (Table 2). Similar findings were observed in subgroups stratified by sexual risk behaviors and prior HIV testing at T0 (eTable 2 in Supplement 2). No significant difference in the CAI with men, multiple male sex partnerships, or uptake of any or specific types of HIV testing was observed between the HIVST-chatbot and the HIVST-OIC groups (Table 3). Within-group changes in study outcomes are presented in eTable 3 in Supplement 2.

**Table 2.** Comparisons of Primary Outcomes Between the HIVST-Chatbot and the HIVST-OIC Groups

Primary outcome	No./total No. (%) of participants		Proportion difference (95% CI), percentage points	RR (95% CI)	ARR (95% CI)	NNT (95% CI)	*P* value
	HIVST-chatbot	HIVST-OIC					
**Intention-to-treat analysis**							
Use of any HIVST	216/266 (81.2)	227/265 (85.7)	−4.5 (−9.8 to 0.8)	0.95 (0.89 to 1.01)	−0.04 (−0.10 to 0.01)	−22.4 (−10.3 to 118.9)	.10
Use of project HIVST	212/266 (79.7)	222/265 (83.8)	−4.1 (−9.6 to 1.4)	0.95 (0.89 to 1.02)	−0.04 (−0.10 to 0.01)	−24.5 (−10.4 to 69.7)	.14
Use of HIVST from other sources	22/266 (8.3)	19/265 (7.2)	1.1 (−2.7 to 4.9)	1.15 (0.70 to 1.89)	0.01 (−0.03 to 0.05)	90.8 (−36.9 to 20.4)	.63
Any counseling support	197/216 (91.2)	142/227 (62.6)	28.7 (22.5 to 34.8)	1.46 (1.33 to 1.60)	0.29 (0.22 to 0.35)	3.5 (2.9 to 4.5)	<.001
Comprehensive counseling	73/216 (33.8)	15/227 (6.6)	27.2 (21.2 to 33.1)	5.11 (3.30 to 7.94)	0.27 (0.21 to 0.33)	3.7 (3.0 to 4.7)	<.001
Simplified counseling	121/216 (56.0)	124/227 (54.6)	1.4 (−6.4 to 9.2)	1.03 (0.89 to 1.18)	0.01 (−0.06 to 0.09)	71.8 (−15.7 to 10.9)	.58
**Complete-case analysis**							
Use of any HIVST	195/240 (81.3)	197/230 (85.7)	−4.4 (−10.0 to 1.2)	0.95 (0.89 to 1.01)	−0.04 (−0.10 to 0.01)	−22.7 (−10.0 to 81.8)	.12
Use of project HIVST	190/240 (79.2)	193/230 (83.9)	−4.8 (−10.6 to 1.1)	0.94 (0.88 to 1.01)	−0.05 (−0.11 to 0.01)	−21.1 (−9.4 to 88.9)	.11
Use of HIVST from other sources	20/240 (8.3)	16/230 (7.0)	1.4 (−2.7 to 5.4)	1.20 (0.70 to 2.04)	0.01 (−0.03 to 0.05)	72.6 (−37.7 to 18.5)	.66
Any counseling support	181/195 (92.8)	120/197 (60.9)	31.9 (25.4 to 38.4)	1.52 (1.38 to 1.68)	0.32 (0.25 to 0.38)	3.1 (2.6 to 3.9)	<.001
Comprehensive counseling	69/195 (35.4)	10/197 (5.1)	30.3 (24.1 to 36.5)	6.97 (4.10 to 11.86)	0.30 (0.24 to 0.37)	3.3 (2.7 to 4.2)	<.001
Simplified counseling	111/195 (56.9)	108/197 (54.8)	2.1 (−6.2 to 10.4)	1.04 (0.90 to 1.20)	0.02 (−0.06 to 0.10)	47.6 (−16.3 to 9.7)	.62

Abbreviations: ARR, absolute risk reduction; HIVST, HIV self-testing; HIVST-chatbot, HIV self-testing service using a chatbot to deliver online real-time instruction and counseling support; HIVST-OIC, HIV self-testing with online real-time support by human administrators; NNT, number needed to treat; RR, relative risk.

**Table 3.** Comparisons of Secondary Outcomes Between the HIVST-Chatbot and HIVST-OIC Groups

Secondary outcome	No./total No. (%) of participants		RR (95% CI)	ARR (95% CI)	NNT (95% CI)	*P* value
	HIVST-chatbot	HIVST-OIC				
**Intention-to-treat analysis**						
CAI with men	124/266 (46.6)	144/265 (54.3)	0.86 (0.72 to 1.02)	−0.08 (−0.16 to 0.01)	−12.95 (−6.17 to 132.13)	.09
Multiple male sex partnerships	120/266 (45.1)	131/265 (49.4)	0.91 (0.76 to 1.09)	−0.04 (−0.13 to 0.04)	−23.14 (−7.81 to 24.02)	.37
Uptake of HIV testing other than HIVST	54/266 (20.3)	70/265 (26.4)	0.77 (0.56 to 1.05)	−0.06 (−0.13 to 0.01)	−16.35 (−7.52 to 93.89)	.11
Uptake of any type of HIV testing	228/266 (85.7)	238/265 (89.8)	0.95 (0.90 to 1.02)	−0.04 (−0.10 to 0.01)	−24.41 (−10.35 to 68.20)	.19
**Complete-case analysis**						
CAI with men	111/240 (46.3)	127/230 (55.2)	0.84 (0.70 to 1.00)	−0.09 (−0.18 to 0.00)	−11.15 (−5.56 to 2654.66)	.06
Multiple male sex partnerships	108/240 (45.0)	114/230 (49.6)	0.91 (0.75 to 1.10)	−0.05 (−0.14 to 0.04)	−21.90 (−7.36 to 22.45)	.37
Uptake of HIV testing other than HIVST	48/240 (20.0)	63/230 (27.4)	0.73 (0.53 to 1.01)	−0.07 (−0.15 to 0.00)	−13.53 (−6.64 to 358.90)	.08
Uptake of any type of HIV testing	207/240 (86.3)	207/230 (90.0)	0.96 (0.90 to 1.02)	−0.04 (−0.10 to 0.02)	−26.67 (−10.44 to 48.02)	.26

Abbreviations: ARR, absolute risk reduction; CAI, condomless anal intercourse; HIVST, HIV self-testing; HIVST-chatbot, HIV self-testing service using a chatbot to deliver online real-time instruction and counseling support; HIVST-OIC, HIV self-testing with online real-time support by human administrators; NNT, number needed to treat; RR, relative risk.

All HIVST users in both the HIVST-chatbot and the HIVST-OIC groups reported their testing results. Two HIVST-chatbot users and 3 HIVST-OIC users received reactive results; all of them were confirmed to be HIV positive and linked to care and treatment. At T1, HIVST-chatbot and HIVST-OIC users reported similarly high levels of engagement with these services (eTable 4 in Supplement 2). We did not receive comments about erroneous recommendations or responses made by the HIVST-chatbot during the study period.

The total cost of the HIVST-chatbot group was lower than that of the HIVST-OIC group (US$27 258.8 vs US$30 885.2). The cost per HIVST user was also lower in the HIVST-chatbot group compared with the HIVST-OIC group (US$139.8 vs US$156.8), as was the cost per HIVST user receiving counseling support (US$150.6 vs US$257.4). The ICER of the HIVST-chatbot was US$1813.2 per additional HIVST user compared with HIVST-OIC, whereas for counseling support the HIVST-chatbot was less costly and more effective and therefore was preferable to the HIVST-OIC (eTables 5 and 6 in Supplement 2).^36^

## Discussion

This RCT contributed to the literature by comparing an innovative HIVST-chatbot with the evidence-based HIVST-OIC and developing new methods to improve HIVST uptake and user support. In line with our hypothesis, the HIVST-chatbot (81.2%) was noninferior to the HIVST-OIC (85.7%) in increasing HIVST uptake. Both HIVST-chatbot and HIVST-OIC ensured good linkage to care. Moreover, more HIVST users in the HIVST-chatbot group received comprehensive counseling support than those in the HIVST-OIC group (33.8% vs 6.6%), whereas a similar proportion of HIVST users used the simplified counseling. Several reasons might explain such differences. The HIVST-OIC users needed to make appointments with the testing administrators to implement the service during working hours, whereas the HIVST-chatbot was available at any time without prior appointment. It was more convenient and flexible to receive comprehensive support provided by the chatbot than from testing administrators. In addition, HIVST-chatbot users might not worry about stigma originating from testing administrators when answering risk assessment in the pretest counseling^37^ because they knew that they were interacting with a nonjudgmental computer program.^38,39,40^ Our economic analysis supported that the HIVST-chatbot was more cost-effective than HIVST-OIC in increasing HIVST uptake and counseling support. Most costs of the HIVST-chatbot were attributed to its development and maintenance. Such costs were less likely to be influenced by the number of users. If more users are allowed to use the chatbot in the future (eg, the HIVST-chatbot becomes publicly available for free), the per-test cost will be largely reduced. However, for HIVST-OIC, the per-test cost was relatively stable regardless of the number of users because the costs mainly originated from the salary of testing administrators. Therefore, it might be easier for HIV testing service providers to implement the HIVST-chatbot, which may increase the currently low HIV testing rate among MSM.

### Limitations

This study has several limitations. First, the HIVST-chatbot and HIVST-OIC were limited to MSM who had access to both a smartphone and WhatsApp. In Hong Kong, smartphone and WhatsApp ownership is high.^25,41^ In this RCT, all MSM who were approached by the research team had access to smartphone and WhatsApp. Second, a 3-arm RCT would be ideal to answer whether HIVST-chatbot or HIVST-OIC are better than no intervention in increasing HIVST uptake. However, there were ethical concerns to withhold a proven effective intervention (HIVST-OIC) to participants in an RCT.^42^ Third, as in most RCTs, our participants were recruited by convenience sampling, and selection bias might exist. Our participants’ baseline characteristics were comparable to local surveillance data.^43^ However, due to the difference in digital literacy and cultures, our findings might not be applicable to other settings. Fourth, the lack of qualitative evaluation data limited our ability to understand user preference and other unexpected findings. Fifth, participants might overreport other HIV testing uptake and underreport sexual risk behaviors due to social desirability.^44,45^ Sixth, we were not able to collect information from refusals. Participants and refusals might have different motivations to receive HIVST, and self-selection bias existed.

## Conclusions

In this RCT, the HIVST-chatbot was noninferior to the evidence-based HIVST-OIC in increasing HIVST uptake and the proportion of HIVST users receiving counseling. With some adaptations, the HIVST-chatbot service can potentially be used in other key populations. Further local and international dissemination and implementation research are needed.

## Notes

Supplement 1.
Trial Protocol

Supplement 2. **eAppendix 1.** Details of the procedures to define the non-inferiority margin and sample size calculation **eAppendix 2.** Development of the HIVST-Chatbot **eFigure.** Architecture of the Chatbot system **eTable 1.** Comparing the baseline characteristics between participants who completed Month 6 follow-up evaluation and those who were lost to follow up **eTable 2.** Comparisons of primary outcomes among different sub-groups of participants between HIVST-Chatbot and HIVST-OIC groups **eTable 3.** Within-group comparisons of primary and secondary outcomes among HIVST-Chatbot and HIVST-OIC groups **eTable 4.** Process evaluation among participants who completed the process evaluation at Month 6 **eTable 5.** Economic evaluation of the HIVST-OIC compared with the HIVST-Chatbot among participants **eTable 6.** Costs items over a 6-month time horizon
eReferences

Supplement 3.
Data Sharing Statement
