# Supplementary material for: Chatbot-Delivered Real-Time Support to Improve HIV Self-Testing Rates: A Randomized Clinical Trial
Source: JAMA Netw Open. 2025 Nov 24;8(11):e2544821. doi: 10.1001/jamanetworkopen.2025.44821 (PMC12645333; doi:10.1001/jamanetworkopen.2025.44821)
Supplement: Supplement 1. — Trial Protocol [file jamanetwopen-e2544821-s001.pdf]

Randomized controlled trial protocol

Evaluating an Innovative HIV Self-Testing Service With Web-Based, Real-Time Counseling Provided by an Artificial Intelligence Chatbot (HIVST-Chatbot) in Increasing HIV Self-Testing Use Among Chinese Men Who Have Sex With Men: Protocol for a Noninferiority Randomized Controlled Trial

Abstract

Background

Counseling supporting HIV self-testing (HIVST) users is essential to ensure support and linkage to care among men who have sex with men (MSM). An HIVST service with online real-time instruction, pre-test and post-test counseling provided by trained administrators (HIVST-OIC) was developed by previous projects. Although the HIVST-OIC was highly effective in increasing HIVST uptake and the proportion of HIVST users receiving counseling along with testing, it required intensive resources to implement and sustain. The service capacity of HIVST-OIC cannot meet the increasing demands of HIVST.

Objectives

The primary objective of this randomized controlled trial (RCT) is to establish whether HIVST-Chatbot, an innovative HIVST service with online real-time instruction and counseling provided by a fully automated Chatbot, would produce effects that are similar to HIVST-OIC in increasing HIVST uptake and proportion of HIVST users receiving counseling alongside testing among MSM within a six-month follow-up period.

Methods

A parallel-group non-inferiority RCT will be conducted. Participants are Chinese-speaking MSM aged 18 years or above with access to live-chat applications. A total of 528 participants will be recruited through multiple sources, including outreach in gay venues, online advertisement, and peer referral. After completing the baseline telephone survey, participants will be randomized evenly into the intervention or control groups. Participants in the intervention group will watch an online video promoting HIVST-Chatbot and receive a free HIVST kit. The Chatbot will contact the participant to implement HIVST and provide standard-of-care and real-time pre-test and post-test counseling, and instruction on how to use the HIVST kit through WhatsApp. Participants in the control group will watch an online video promoting HIVST-OIC and receive a free HIVST kit in the same manner. Upon appointment, a trained testing administrator will implement HIVST and provide standard-of-care and real-time pre-test and post-test counseling and instruction on how to use the HIVST kit through live-chat applications. All participants will complete a telephone follow-up survey six months after the baseline. The primary outcomes are HIVST uptake and the proportion of HIVST users receiving counseling support along with testing in the past six months measured at Month 6. Secondary outcomes include sexual risk behaviors and uptake of HIV testing other than HIVST during the follow-up period. Intention-to-treat analysis will be used.

Results

Recruitment and enrollment of participants will start in April 2023.

Conclusion

This study will generate important research and policy implications regarding using Chatbot in HIVST services. If HIVST-Chatbot is proven non-inferior to HIVST-OIC, it can be easily integrated into existing HIVST services in Hong Kong. The coverage of HIV testing and the level of support to HIVST users will be increased.

Trial Registration

ClinicalTrial.gov (number NCT05796622);
https://clinicaltrials.gov/ct2/show/NCT05796622?term=NCT05796622&draw=2&rank=1

Keywords: HIV self-testing, Chatbot, men who have sex with men, counseling, non-inferiority randomized controlled trial

Introduction

Background

Globally, the HIV epidemic among men who have sex with men (MSM) remains out of control.[1] High coverage of HIV testing among the at-risk population is the first and crucial step to achieving the 90-90-90 targets set by the joint United Nations Program on HIV/AIDS (UNAIDS).[2] Therefore, international health authorities recommend that MSM take up HIV testing every six months.[3,4] However, despite the high risk of HIV infection among MSM in the Hong Kong Special Administrative Region (SAR) of China (HIV prevalence: 6.54% in 2017), their HIV testing rate remained inadequate (65% in the past year).[5]

HIV self-testing (HIVST) could remove the obstacles of HIV testing faced by MSM, such as inconvenience and perceived stigma from service providers.[6] A systematic review and meta-analysis suggested that HIVST could significantly increase the frequency and coverage of HIV testing among MSM.[7] Therefore, the World Health Organization (WHO) strongly recommends that HIVST should be offered as an additional approach to existing HIV testing services.[8] MSM in Hong Kong mainly relied on facility-based HIV testing and counseling provided by community-based organizations (CBO) and governmental clinics.[9] During the COVID-19 pandemic, facility-based HIV testing services provided by CBOs and governmental clinics were suspended or closed in Hong Kong.[10] However, the availability of HIVST kits in Hong Kong was not affected by the pandemic. An increasing demand for HIVST among MSM was observed in Hong Kong.[11]

Pre-test and post-test counseling are essential for HIVST, facilitating linkage to care and behavioral change among HIVST users. In standard-of-care HIV testing and counseling (HTC), the pre-testing counseling involves assessing the individual's risks, providing information about HIV testing, asserting the user's right to refuse the test, and informing the user about the possibility of beneficial disclosure of sero-status status, as well as providing preventive information and material.[12] Post-test counseling entails interpreting testing results, offering psychological support to individuals with positive results, facilitating beneficial disclosure of positive sero-status, and referring individuals for further care, treatment, and support services. Additionally, preventive information and material are provided for those with negative test results.[12] Despite the advantages of HIVST, HIVST users may skip pre-testing or post-test counseling. There are concerns about incorrect procedures, inadequate support for users, and potential issues related to the linkage of care.[13] A meta-analysis study examined that HIVST reduced linkage to care by 47% comparing facility-based HIV testing due to the lack of counseling support.[14]

In previous projects, an evidence-based HIVST service was developed for MSM in Hong Kong, which included online promotion of HIVST, delivery of free HIVST kits, and provision of online real-time instruction and standard-of-care pre-test/post-test counseling via live chat application (HIVST-OIC).[15] The HIVST-OIC is highly effective in increasing HIVST uptake and the proportion of HIVST users receiving counseling along with testing. A randomized controlled trial (RCT) showed that after being offered HIVST-OIC, 87.9% of participants took up HIVST, and all HIVST users received counseling. Moreover, the HIVST-OIC was effective in reducing sexual risk behaviors among users. The Centre for Disease Control and Prevention (CDC) listed HIVST-OIC as an evidence-based intervention and best practice.[16] However, there are issues during the implementation, as HIVST-OIC requires intensive resources. Therefore, it is difficult to increase the service capacity of HIVST-OIC, resulting in a significant gap in implementation.

A Chatbot is a computerized program that can automatically select and provide different paths of intervention according to participants' responses. The Chatbot can offer personalized, engaging, and on-demand health communication. With the advance in artificial intelligence, a Chatbot can learn from previous human-machine interactions to increase the accuracy and quality of future interactions.[17] A recent systematic review demonstrated the feasibility and effectiveness of Chatbot in promoting healthy lifestyles, smoking

cessation, treatment adherence, and substance misuse.[18] The Chatbot is potentially useful for delivering real-time counseling to support HIVST users. To our knowledge, this approach is novel.

Objectives

This study aims to demonstrate whether an innovative HIVST service with online real-time instruction and counseling provided by a fully automated Chatbot (HIVST-Chatbot) is as efficacious, if not more efficacious, as HIVST-OIC in increasing HIVST uptake and the proportion of HIVST users receiving counseling alongside testing among MSM in Hong Kong.

The primary objective is to establish whether HIVST-Chatbot would produce effects similar to HIVST-OIC in increasing HIVST uptake and the proportion of HIVST users receiving counseling alongside testing within a six-month follow-up period. We hypothesize that HIVST-Chatbot would be statistically non-inferior to HIVST-OIC in increasing HIVST uptake and the proportion of HIVST users receiving counseling alongside testing. The non-inferiority margin is set as 10% (HIVST-Chatbot is no worse than 10% less than that of the HIVST-OIC). The secondary objectives are to compare HIVST-Chatbot with HIVST-OIC on the prevalence of condomless anal intercourse (CAI), multiple male sex partnerships, and uptake of HIV testing other than HIVST at Month 6.

Methods

Study design

A parallel-group non-inferiority randomized controlled trial will be conducted. A total of 528 participants are randomized 1:1 to either the intervention group (n=264) or the control group (n=264). We will promote and implement HIVST-Chatbot and HIVST-OIC in the intervention group and control group, respectively. A telephone follow-up evaluation will be conducted six months after the baseline survey by blinded interviewers. The study was registered at ClinicalTrial.gov (number NCT05796622). A flowchart diagram is shown in Figure 1.

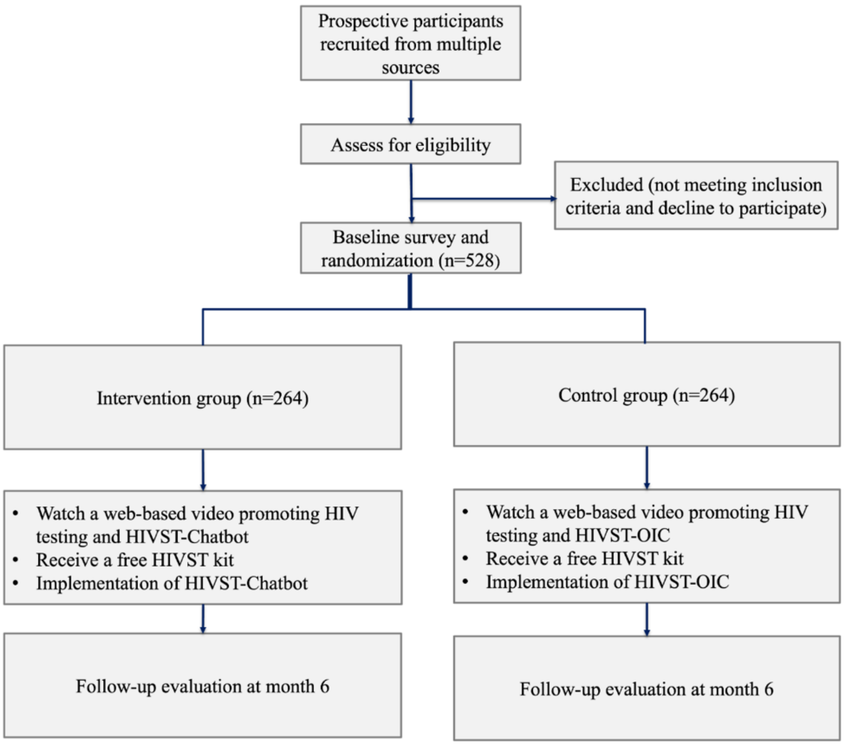

Figure 1. Flowchart diagram for the non-inferiority randomized controlled trial

Participants and recruitments

Inclusion criteria are 1) Hong Kong Chinese-speaking men aged at least 18 years old, 2) anal intercourse with at

least one man within the last six months, 3) willing to provide contact information for follow-up at Month 6, (4) having access to WhatsApp, and 5) without intention to leave Hong Kong for one month consecutively in the next six months. Those who have been diagnosed as HIV-positive will be excluded.

Participants will be recruited through multiple sources. Upon obtaining the approval of the owners, trained and experienced fieldworkers will approach prospective MSM participants in gay bars and saunas at different timeslots during weekdays and weekends. They will brief prospective participants about the study details and give them an information sheet. The research team will also conduct online outreach by posting study information periodically as discussion topics on gay websites with the highest traffic in Hong Kong. If prospective participants are interested in this study, they can contact the research team through private messaging, WhatsApp, telephone, or email. Recruitment will be supplemented by peer referrals. Participants will be guaranteed anonymity during the study, the right to quit at any time, and that refusal will have no consequences. Verbal instead of written informed consent will be obtained to allow participants to maintain anonymity. Fieldworkers will sign a form pledging that the participants have been fully informed about the study. Similar methods to obtain informed consent have been commonly used in interventional studies targeting MSM in Hong Kong.[15,19-21] Multiple forms of contact information will be obtained to make an appointment for a baseline telephone survey, which takes about 20 minutes to complete. Another telephone follow-up survey will be conducted six months after the completion of the baseline survey. A supermarket coupon (HK$50) will be mailed to an address provided by the participants upon completion of each survey. Ethics approval was obtained from the Survey and Behavioral Research Ethics Committee of the Chinese University of Hong Kong (Ref#: SBRE-22-0488).

Sample size planning

The target sample size is 528 (264 per group). Our previous study showed that about 90% of MSM used HIVST after exposure to HIVST-OIC promotion and implementation.[15] We conservatively assume that 80% of participants in the control group will take up HIVST during the project period. A non-inferiority margin of between-group difference of this primary outcome is set at 10% (no worse than 10% less than that of the control group). A sample size of 198 per group will be sufficient to confer 80% power at the one-sided significance level of 0.05 (PASS 11.0). Given the 25% drop-out rate during the follow-up period,[22] 264 participants per group (528 total) will be needed for the study.

The baseline survey and random allocation process

Participants will be randomized evenly to either the intervention or control groups. Computer-generated random allocation codes will be produced and sealed in opaque envelopes by a research staff member without involvement in recruitment or baseline survey. One envelope will be drawn and opened by the fieldworkers. They will then inform the participant which group he is assigned to. Block randomization with a block size of eight will be used.

The control group

Online promotion of HIVST-OIC

Participants will watch a 7-minute online video promoting HIV testing in general and HIVST-OIC developed by previous projects.[15,20] The online video is based on the constructs of perceived benefits and perceived barriers of the Health Belief Model.[23] The video has two parts. The first part is to promote HIV testing in general. A local MSM introduces the potential benefits of taking up HIV testing in general (e.g., help to detect HIV infection earlier, increase trust between sex partners and reduce psychological burdens) and the WHO recommendation for MSM with risk behaviors to take up HIV testing every six months. In the second part, the same MSM narratively discusses the benefits and barriers of HIVST-OIC, demonstrates its procedures, and emphasizes the availability of real-time online support.

Signing up for HIVST-OIC

After completing watching the online video, participants are asked to select their preferable HIVST kits (oral fluid-based or blood-based), choose a service model (comprehensive version with real-time instruction, pre-test, and post-test counseling, or simplified version with real-time post-test counseling only), and fill in their contact information and address to receive the HIVST kits. Participants can receive a free HIVST kit through rapid courier service, mail it in a plain envelope, or pick it up at collaborative CBO or the research office. Participants can make an appointment with trained HIV testing administrators to implement HIVST-OIC after receiving the HIVST kit. OraQuick ® in-home test kit (OraSure Technologies, Inc.; sensitivity: 91.7%; specificity: 99.9%) and BioSure ® HIV self-test kit (BioSure, Inc.; sensitivity: 99.7%; specificity: 99.9%) will be used in this study.

Implementation of HIVST-OIC

The implementation will follow the same procedures reported in previous studies.[15,20] Through live-chat applications (e.g., Line, WhatsApp, Skype), an experienced HIV testing administrator will guide the participants through self-test procedures on-screen and in real-time. Participants are guaranteed anonymity, and no taping will be made. Users may elect to refrain from displaying their faces on the screen. Standard-of-care pre-test and post-test counseling will be provided if the user chooses the comprehensive version. The administrator will also explain how to use the HIVST kits and send the user a demonstration video if needed. The entire procedure of the comprehensive version takes about 60 minutes to complete, which is comparable to the time required for facility-based HIV testing and counseling. If the user prefers a simplified version, pre-test counseling will be replaced by online pre-test information (e.g., window period of HIVST, types of sexual behaviors with a high risk of HIV transmission). Users can access the demonstration video and standard-of-care real-time counseling. The simplified version will take about 20 minutes to complete. Users screened to be positive will be given immediate psychological support and explained about the need to take up free confirmatory HIV antibody testing offered by the Department of Health. If necessary, the collaborative CBO staff will accompany them to the CBO or Department of Health.

The intervention group

Online promotion of HIVST-Chatbot

Participants in the intervention group will watch an online video promoting HIV testing in general and HIVST-Chatbot. The first part of the online video promoting HIV testing generally is the same as the one for the control group. In the second part, the same MSM narratively discusses the advantages of HIVST-Chatbot (no need to wait for an appointment, operates 24 hours a day, artificial intelligence) and the availability of real-time support from Chatbot and supporting staff (if users receive positive screening results). The video will also demonstrate the procedures of the HIVST-Chatbot. Project staff will help the participants connect to the Chatbot. Participants are guaranteed anonymity during the implementation.

Signing up for HIVST-Chatbot

Participants will receive a free HIVST kit in the same manner. The Chatbot will contact the participants to confirm receipt of the kits. Participants can get the Chatbot to implement HIVST without a prior appointment.

Implementation of HIVST-Chatbot

1) Pre-test counseling

When users contact the Chatbot for implementation, the Chatbot will provide standard-of-care pre-test and post-test counseling covering key components listed in the Quality Assurance Guideline on HIV Testing and Counseling Services in the format of text messages or videos.[24] The workflow of the HIVST-Chatbot is presented in Figure 2.

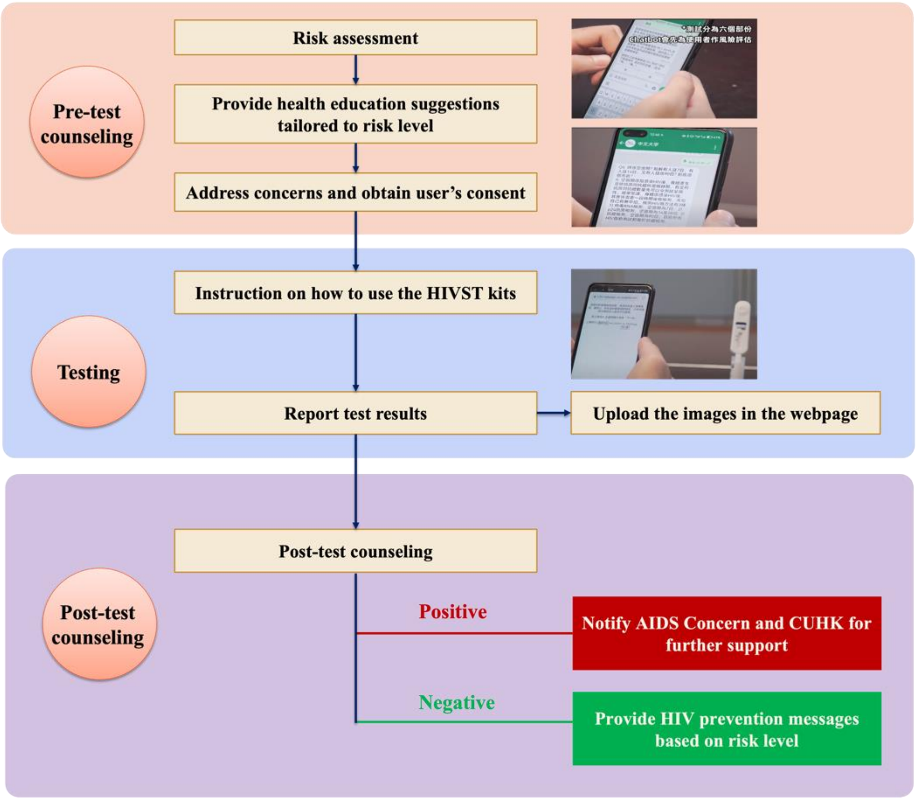

Figure 2. The workflow of the HIVST-Chatbot

a) Risk assessment: The Chatbot will ask some standard questions to assess users' HIV risk (e.g., injective drug use, use of condoms and alcohol/drug during sexual behaviors, and use of pre-exposure prophylaxis) and show the response categories. After users complete the questions, the Chatbot will interpret their responses and define their risk level. The questions and definition of risk level are identical to those used by facility-based HIV testing and counseling services in Hong Kong (multimedia appendix 1).

b) Providing health education advice tailored to risk level: The Chatbot will inform users about their risk level and explain the rationale. For high-risk users, the Chatbot will prepare them for potential positive results. It will explain the implication of positive screening results and follow-up procedures and emphasize that the staff of collaborative CBO will provide immediate support for users receiving positive testing results from 10:00 am to 9:00 pm (Monday to Friday). These users are advised to perform HIVST within such a timeframe. For users without high risk, advice on maintaining safe sex practices is provided. Users can skip parts a) and b). If they prefer to skip these parts, standard pre-test information will be sent to them (e.g., window period of HIVST, types of sexual behaviors with a high risk of HIV transmission).

c) Addressing concerns and obtaining consent for HIVST. Users can choose one or more references from a list of common concerns identified by literature review and interviews of local MSM and service providers. In case their problems are not included in the list, the participant can raise them verbally or in written text. The Chatbot will interpret users' input, extract keywords, retrieve relevant information from the knowledge graph, and prepare a response in the text to the participants. Ultimately, the Chatbot will ask whether users are ready to perform HIVST.

2) Instruction on how to use the HIVST kit

The Chatbot will play a pre-recorded online demonstration video showing the procedures of using an oral fluid-based/blood-based HIVST kit. Users can watch the demonstration video repeatedly. They can also skip it if they are familiar with the procedures.

3) Reporting testing results

The Chatbot will show examples of positive, negative, and invalid results to help the users interpret them. Users will report their results by answering a multiple-choice question.

The Chatbot will ask the users to take a photo of their testing result and upload it to a secure webpage for verification.

4) Post-test counseling

For users receiving negative testing results, the Chatbot will explain that the user is not infected unless the test was taken within the window period, remind them about the risk of HIV infection, and provide advice for safe sex practices. Ultimately, the Chatbot will emphasize the need for regular HIV testing.

For users receiving positive testing results, the Chatbot will automatically notify a designated supporting staff from the collaborative CBO after obtaining users' consent. The supporting staff will follow up with the user as soon as possible. The Chatbot will explain: a) this is a preliminary positive test result, b) further confirmatory testing in the Department of Health is required, c) a staff from collaborative CBO is already notified and will follow up with them as soon as possible, and d) emphasize that HIV treatment is very effective. Hotline numbers for 24-hour emotional support and the collaborative CBO will also be provided. Our project staff may accompany them to visit the collaborative CBO or the Department of Health if desired.

Development of the HIVST-Chatbot

The architecture of the Chatbot system

We adopted the WhatsApp platform to implement the Chatbot. The Chatbot is integrated with WhatsApp through its public Web API services. Participants' messages are sent to WhatsApp's instant messaging server and a separately constructed Chatbot system (an administrative system and the Chatbot). The Chatbot system processes and sends a message back to the WhatsApp instant messaging server. Finally, the users can view the message sent by the Chatbot. The entire process spans less than a second without a sluggish feel for users.

The Chatbot system contains three modules.

1) Dialogue management module: The dialogue system records all conversations between users and the Chatbot. The system can extract context information such as basic statistics of users' activities and prior interaction between a user and the Chatbot. The natural language processing module analyzes the text content of each message. Then, the message is forwarded to trigger specific actions based on preprogrammed rules. For example, when a user sends a message containing a specific keyword, the Chatbot immediately responds with information that corresponds to that keyword. In addition, the module initiates new conversations based on the preprogrammed intervention plan.

2) User management module: This module records detailed information about user conversations. Administrators can link users' WhatsApp numbers with the Chatbot and review the progress of the intervention delivery (e.g., number of completed sessions, disconnection between users and the Chatbot).

3) Multi-media management module: The Chatbot system supports image uploading, sending, and updating through this module to allow image and video exchange.

Preparation of knowledge graph

To build a system that addresses various personal and relevant concerns related to HIV, HIV testing, and HIVST, we created a knowledge graph linking questions and responses. We conducted in-depth interviews with 10 local MSM to crowdsource concerns related to HIV, HIV testing, and HIVST. We also interviewed experienced HIV testing administrators, CBO workers, and experts in HIV prevention to obtain responses addressing these concerns. The knowledge graph will be used to set rules for the Chatbot and train it to get familiar with and identify commonly encountered concerns/misinformation and how to address them through machine learning.

The knowledge graph will evolve over time with more prior interactions during the intervention period.

Conversation mechanism

1) The Chatbot uses a mixture of techniques so that a dialogue is not merely rule-based, but it can also learn from a big pool of prior conversation patterns through machine learning. The Chatbot resolves the recent conversation to create a plan for a suitable response. Relevant information is retrieved and used for the preparation of a reply. A persuasion template is selected in accordance with the current stance of the conversation and the perceived user characteristics by the Chatbot. The pool of prior conversation patterns will grow bigger after extended usage, and the variety of responses will increase accordingly.

2) In case there is no relevant response to participants' input, the Chatbot will respond by saying, "I am not confident about an answer to your question." The Chatbot will suggest a new conversation topic after three off-topic inputs from the user.

3) In case the participants want to know more about one topic. The Chatbot will respond differently within the knowledge graph to the same topic. Upon request, the Chatbot can also repeat the response.

4) In case the conversation between Chatbot and the participants is interrupted. The Chatbot will cancel participants' incomplete input and invite them to input again.

5) The Chatbot will document prior conversations with the users and avoid responding repetitively to users' questions.

Pilot testing and refinement

We purposely recruited 30 MSM who were HIV negative or unknown sero-status to use the HIVST-Chatbot. With informed consent, the research team retrieved and reviewed users' interactions with the Chatbot. Feedback was also collected from these users. The pilot study showed that the Chatbot was running smoothly. Users were satisfied with the performance and interface of the Chatbot.

Measurements

Primary outcomes

The primary outcomes of this study are self-reported uptake of any HIVST at Month 6 and the proportion of HIVST users self-reported having received counseling along with HIVST during the follow-up period. The Chatbot will accurately document the number of HIVST-Chatbot use, while HIV testing administrators will record the number of HIVST-OIC performed.

Secondary outcomes

1) Condomless anal intercourse with men and multiple male sex partnerships in the past six months measured at Month 6.

2) Uptake of other types of HIV testing in the past six months measured at Month 6. They are a) HIV testing at CBOs in Hong Kong, b) HIV testing at public hospitals/clinics in Hong Kong, c) HIV testing at private hospitals/clinics in Hong Kong, d) HIV testing at other organizations in Hong Kong, and e) HIV testing in places other than Hong Kong.

Baseline background characteristics

Information on social demographics (i.e., age, current relationship status, education level, income), sexual orientation, history of HIV testing, COVID-19, and other sexually transmitted infections will be collected. In addition, participants are asked to report the use of pre-exposure prophylaxis and different HIV or sexually transmitted infection prevention services (receiving free condoms, peer education and pamphlets, and attending workshops).

Process evaluation

At Month 6, participants will be asked about their level of satisfaction with the promotion and implementation of HIVST-Chatbot/HIVST-OIC.

Statistical analysis

Intention-to-treat analysis will be performed. Multiple imputations will handle missing values at Month 6. Markov Chain Monte Carlo method will be used to impute data with the arbitrary pattern of missing values, while monotone methods are used to impute data having a monotone pattern of missing values. Predictors included baseline background characteristics and baseline values of these outcomes. The relative risk reduction, absolute risk reduction, and number needed to treat, and their 95% confidence interval will be calculated using Excel. The chi-square tests will inspect between-group balances of potential confounders at baseline. Adjustments will be made if any potential confounders show $P<.05$ in the comparisons. SPSS 26.0 for Windows (SPSS Inc., Chicago, IL) will be used for all analyses; statistical significance is set at the $P<.05$ level (one-sided).

Results

Study recruitment will start in April 2023, and data collection is expected to be concluded in January 2024. No results are available as of manuscript preparation.

Discussion

The COVID-19 pandemic and its control measures (e.g., lockdown, closure of business, physical distancing) have direct and negative impacts on HIV testing service utilization among MSM, especially for facility-based HIV testing services.[25-28] Across countries, health authorities have been actively promoting HIVST to mitigate such negative impacts.[29-31] As a result, there was an increase in HIVST use among MSM.[11] Such growth may continue even in the post-pandemic era. Therefore, ensuring sufficient support and linkage to care for the increasing number of MSM HIVST users becomes more important. Although evidence-based HIVST services existed (e.g., HIVST-OIC), most required intensive resources to implement and maintain. Such constraints limited their public health impacts. To our knowledge, this is the first attempt to apply an artificial intelligence Chatbot to deliver real-time instruction and counseling support to HIVST users. This RCT will compare the efficacy of the HIVST-Chatbot with an evidence-based HIVST service, the HIVST-OIC, in increasing HIVST uptake and the proportion of HIVST users receiving counseling along with the testing. If HIVST-Chatbot is proven non-inferior to HIVST-OIC in improving HIVST uptake and coverage of counseling among users, it may address the existing HIVST service gaps. Since HIVST-Chatbot is fully-automated and requires fewer resources to implement and maintain, it can be promoted and implemented by governmental organizations and CBOs in Hong Kong as an alternative approach to HIVST-OIC. The HIVST-Chatbot has the potential to overcome the barriers to using HIVST (e.g., concerns about lack of support and missing linkage to care), improve overall HIV testing coverage, and reduce the HIV burden among MSM.

Although the trial will yield essential insights for HIVST program planning and policymaking, it has some limitations. First, the use of HIV testing other than HIVST-Chatbot or HIVST-OIC is self-reported. Such responses may be over-reported due to social desirability. Second, similar to most interventional studies,[15,19-21] participants will be recruited through convenient sampling. Cautious should be taken when generalizing the findings to MSM in Hong Kong. Third, collecting characteristics of MSM who refuse to participate in the study is difficult. Selection bias may exist.

Conclusions

This study will generate important research and policy implications regarding using Chatbot in HIVST services. If HIVST-Chatbot is proven non-inferior to HIVST-OIC, it can be easily integrated into HIVST services provided by the governmental organization and CBOs in Hong Kong. The coverage of HIV testing and the level of support to HIVST users will be vastly increased.

References:

1. Dong MJ, Peng B, Liu ZF, et al. The prevalence of HIV among MSM in China: a large-scale systematic analysis. *BMC infectious diseases*. Nov 27 2019;19(1):1000. doi:10.1186/s12879-019-4559-1
2. Tsui H, Lau JT, Xiang W, Gu J, Wang Z. Should associations between HIV-related risk perceptions and behaviors or intentions be positive or negative? *PloS one*. 2012;7(12):e52124. doi:10.1371/journal.pone.0052124
3. Gu J, Bai Y, Lau JT, et al. Social environmental factors and condom use among female injection drug users who are sex workers in China. *AIDS and behavior*. Feb 2014;18 Suppl 2:S181-91. doi:10.1007/s10461-013-0434-z
4. Lau JT, Gu J, Tsui HY, et al. Prevalence and associated factors of condom use during commercial sex by female sex workers who were or were not injecting drug users in China. *Sexual health*. Sep 2012;9(4):368-76. doi:10.1071/SH11108
5. Lau JT, Cheng Y, Gu J, et al. Suicides in a mega-size factory in China: poor mental health among young migrant workers in China. *Occupational and environmental medicine*. Jul 2012;69(7):526. doi:10.1136/oemed-2011-100593
6. Hamilton A, Shin S, Taggart T, et al. HIV testing barriers and intervention strategies among men, transgender women, female sex workers and incarcerated persons in the Caribbean: a systematic review. *Sex Transm Infect*. May 2020;96(3):189-196. doi:10.1136/sextrans-2018-053932
7. Johnson CC, Kennedy C, Fonner V, et al. Examining the effects of HIV self-testing compared to standard HIV testing services: a systematic review and meta-analysis. *J Int AIDS Soc*. May 15 2017;20(1):21594. doi:10.7448/ias.20.1.21594
8. World Health Organization. The WHO guideline on HIV Self-testing and Partner Notification. Available at: http://www.who.int/hiv/pub/vct/hiv-self-testing-guidelines/en/. 2016;
9. Lau JT, Yeung NC, Mui LW, Tsui HY, Gu J. A simple new method to triangulate self-reported risk behavior data--the bean method. *Sexually transmitted diseases*. Sep 2011;38(9):788-92. doi:10.1097/OLQ.0b013e318218cc66
10. Suen YT, Chidgey A. Disruption of HIV Service Provision and Response in Hong Kong During COVID-19: Issues of Privacy and Space. *J Int Assoc Pro Aids*. Jan-Dec 2021;20doi:Artn 23259582211059588 10.1177/23259582211059588
11. Wang Z, Chan PSF, Xin M, et al. An online intervention promoting HIV testing services utilization among Chinese men who have sex with men during the COVID-19 pandemic: a quasi-experimental study. *AIDS and behavior*. 2023;
12. World Health Organization. AIDS and sexually transmitted diseases. Accessed 19 October, 2022. https://www.emro.who.int/asd/about/testing-counselling.html
13. Hamilton A, Thompson N, Choko AT, et al. HIV Self-Testing Uptake and Intervention Strategies Among Men in Sub-Saharan Africa: A Systematic Review. *Frontiers in Public Health*. Feb 2021;9594298. doi:10.3389/fpubh.2021.594298
14. Witzel TC, Eshun-Wilson I, Jamil MS, et al. Comparing the effects of HIV self-testing to standard HIV testing for key populations: a systematic review and meta-analysis. *BMC Med*. Dec 3 2020;18(1):381. doi:10.1186/s12916-020-01835-z
15. Wang Z, Lau JTF, Ip M, et al. A Randomized Controlled Trial Evaluating Efficacy of Promoting a Home-Based HIV Self-Testing with Online Counseling on Increasing HIV Testing Among Men Who Have Sex with Men. *AIDS Behav*. Jan 2018;22(1):190-201. doi:10.1007/s10461-017-1887-2
16. Center for Disease Control and Prevention. HOME-BASED HIV SELF-TESTING WITH ONLINE

INSTRUCTION AND COUNSELING (HIVST-OIC). Available at: https://www.cdc.gov/hiv/pdf/research/interventionresearch/compendium/si/cdc-hiv-Home_Based_HIV_Self_Testing_Online_SI_EBI.pdf. Accessed on July 23, 2020. 2020;

17. Fadhil A, Gabrielli S. Addressing challenges in promoting healthy lifestyles: the al-chatbot approach. PervasiveHealth '17: Proceedings of the 11th EAI International Conference on Pervasive Computing Technologies for Healthcare May 2017 https://doi.org/10.1145/3154862.3154914. 2017:261-265.

18. Aggarwal A, Tam CC, Wu D, Li X, QIao S. Artificial Intelligence (AI)-based Chatbots in Promoting Health Behavioral Changes: A Systematic Review. medRxiv preprint. Available at: https://www.medrxiv.org/content/10.1101/2022.07.05.22277263v1. Accessed on November 30, 2022. 2022;

19. Wang Z, Lau JTF, Ip TKM, et al. Two Web-Based and Theory-Based Interventions With and Without Brief Motivational Interviewing in the Promotion of Human Papillomavirus Vaccination Among Chinese Men Who Have Sex With Men: Randomized Controlled Trial. *Journal of medical Internet research*. Feb 2 2021;23(2):e21465. doi:10.2196/21465

20. Chan PS, Chidgey A, Lau J, Ip M, Lau JTF, Wang Z. Effectiveness of a Novel HIV Self-Testing Service with Online Real-Time Counseling Support (HIVST-Online) in Increasing HIV Testing Rate and Repeated HIV Testing among Men Who Have Sex with Men in Hong Kong: Results of a Pilot Implementation Project. *International journal of environmental research and public health*. Jan 15 2021;18(2)doi:10.3390/ijerph18020729

21. Wang Z, Fang Y, Chan PS, et al. Effectiveness of a Community-Based Organization-Private Clinic Service Model in Promoting Human Papillomavirus Vaccination among Chinese Men Who Have Sex with Men. *Vaccines (Basel)*. Oct 20 2021;9(11)doi:10.3390/vaccines9111218

22. Lau JT, Lee AL, Tse WS, et al. A Randomized Control Trial for Evaluating Efficacies of Two Online Cognitive Interventions With and Without Fear-Appeal Imagery Approaches in Preventing Unprotected Anal Sex Among Chinese Men Who Have Sex with Men. *AIDS and behavior*. Jan 22 2016;doi:10.1007/s10461-015-1263-z

23. Janz NK, Becker MH. The Health Belief Model: a decade later. *Health education quarterly*. Spring 1984;11(1):1-47. doi:10.1177/109019818401100101

24. Hong Kong Advisory Council on AIDS. Quality Assurance Guidelines on HIV Voluntary Counselling and Testing Services in Community Settings. Available at: https://www.atf.gov.hk/english/forms/qa_setting.html. Accessed on July 22, 2020. 2016;

25. Booton RD, Fu GF, MacGregor L, et al. The impact of disruptions due to COVID-19 on HIV transmission and control among men who have sex with men in China. *J Int Aids Soc*. Apr 2021;24(4)doi:ARTN e25697
10.1002/jia2.25697

26. Zhang KC, Chen SY, Chan PSF, et al. Changes in HIV Testing Utilization Among Chinese Men Who Have Sex With Men During the COVID-19 Pandemic in Shenzhen, China: An Observational Prospective Cohort Study. *Front Med-Lausanne*. Jun 9 2022;9doi:ARTN 842121
10.3389/fmed.2022.842121

27. Suen YT, Chan RCH, Wong EMY. An exploratory study of factors associated with difficulties in accessing HIV services during the COVID-19 pandemic among Chinese gay and bisexual men in Hong Kong. *Int J Infect Dis*. May 2021;106:358-362. doi:10.1016/j.ijid.2021.04.005

28. Zhang KC, Fang Y, Cao H, et al. The Impacts of the COVID-19 Pandemic on HIV Testing Utilization Among Men Who Have Sex With Men in China: Cross-sectional Online Survey. *JMIR public health and surveillance*. May 25 2022;8(5):e30070. doi:10.2196/30070

29. van Kesteren NM, Hospers HJ, Kok G. Sexual risk behavior among HIV-positive men who have sex with

men: a literature review. *Patient education and counseling*. Jan 2007;65(1):5-20. doi:10.1016/j.pec.2006.09.003

30. Hecht J, Sanchez T, Sullivan PS, DiNenno EA, Cramer N, Delaney KP. Increasing Access to HIV Testing Through Direct-to-Consumer HIV Self-Test Distribution - United States, March 31, 2020-March 30, 2021. *Mmwr-Morbid Mortal W*. Sep 24 2021;70(38):1322-1325.

31. Maatouk I, El Nakib M, Assi M, et al. Community-led HIV self-testing for men who have sex with men in Lebanon: lessons learned and impact of COVID-19. *Health Res Policy Sy*. Apr 21 2021;19(Suppl)doi:ARTN 50 10.1186/s12961-021-00709-x
