# Supplementary material for: Chatbot-Delivered Real-Time Support to Improve HIV Self-Testing Rates: A Randomized Clinical Trial
Source: JAMA Netw Open. 2025 Nov 24;8(11):e2544821. doi: 10.1001/jamanetworkopen.2025.44821 (PMC12645333; doi:10.1001/jamanetworkopen.2025.44821)
Supplement: Supplement 2. — eAppendix 1. Details of the procedures to define the non-inferiority margin and sample size calculation eAppendix 2. Development of the HIVST-Chatbot eFigure. Architecture of the Chatbot system eTable 1. Comparing the baseline characteristics between participants who completed Month 6 follow-up evaluation and those who were lost to follow up eTable 2. Comparisons of primary outcomes among different sub-groups of participants between HIVST-Chatbot and HIVST-OIC groups eTable 3. Within-group comparisons of primary and secondary outcomes among HIVST-Chatbot and HIVST-OIC groups eTable 4. Process evaluation among participants who completed the process evaluation at Month 6 eTable 5. Economic evaluation of the HIVST-OIC compared with the HIVST-Chatbot among participants eTable 6. Costs items over a 6-month time horizon eReferences [file jamanetwopen-e2544821-s002.pdf]

Supplemental Online Content

Chen S, Yu F-y, Fang Y, et al. Chatbot-delivered real-time support to improve HIV self-testing rates: a randomized clinical trial. *JAMA Netw Open or JAMA Health Forum*. 2025;8(11):e2544821. doi:10.1001/jamanetworkopen.2025.44821

eAppendix 1. Details of the procedures to define the non-inferiority margin and sample size calculation
eAppendix 2. Development of the HIVST-Chatbot
eFigure 1. Architecture of the Chatbot system
eTable 1. Comparing the baseline characteristics between participants who completed Month 6 follow-up evaluation and those who were lost to follow up
eTable 2. Comparisons of primary outcomes among different sub-groups of participants between HIVST-Chatbot and HIVST-OIC groups
eTable 3. Within-group comparisons of primary and secondary outcomes among HIVST-Chatbot and HIVST-OIC groups
eTable 4. Process evaluation among participants who completed the process evaluation at Month 6
eTable 5. Economic evaluation of the HIVST-OIC compared with the HIVST-Chatbot among participants
eTable 6. Costs items over a 6-month time horizon
eReferences

This supplemental material has been provided by the authors to give readers additional information about their work.

© 2025 Chen S et al. *JAMA Network Open.*

eAppendix 1 Details of the procedures to define the non-inferiority margin and sample size calculation

In our previous randomized controlled trial, 87.9% of men who have sex with men (MSM) in Hong Kong utilized HIV self-testing (HIVST) within a 6-month study period after exposure the promotion and implementation of human administer-delivered real-time counseling support (HIVST-OIC).[1] For planning purpose, we conservatively estimated about 80% of participants will take up HIVST after exposure to the same health promotion within the same follow-up period. We held a panel discussion with stakeholders, including government officials from Centre for Health Protection, leaders of non-governmental organizations (NGOs) providing HIV testing services, and researchers working in HIV prevention to understand their expectation about the chatbot-delivered real-time counseling support (HIVST-Chatbot). The panel reached the following consensus: (1) it was acceptable that the HIVST-Chatbot would lead to lower HIVST uptake comparing to HIVST-OIC as a trade-off for cost-saving and sustainability, (2) the HIVST-Chatbot should have a better performance than previous HIVST promotion programs in Hong Kong (e.g., 54.3% in a multimedia campaign launched by an NGO)[2] and mainland China (32.7-54.7%),[3-5] with an absolute improvement of at least 15%. Therefore, the panel expected at least 70% of the participants after exposure to HIVST-Chatbot would take up HIVST. The non-inferiority margin was hence defined as 10% (70% in the HIVST-Chatbot vs. 80% in the HIVST-OIC).

Based on these assumptions, the sample size was calculated as follows: assuming 80% uptake in the HIVST-OIC group at T1 and applying a 10% non-inferiority margin (requiring at least 70% uptake in the HIVST-Chatbot group),[6,7] 198 participants per group were needed to achieve 80% power at a two-sided α=0.05 (PASS 11.0; NCSS). Considering an anticipated dropout rate of 25% at six months (T1), 264 participants per group were required.

© 2025 Chen S et al. *JAMA Network Open.*

eAppendix 2 Development of the HIVST-Chatbot

1) Architecture of the chatbot

Our HIVST-Chatbot is a natural language processing (NLP)-based chatbot. It is not publicly available at this stage, only participants in the HIVST-Chatbot group had access to the chatbot during the project period. We integrated the chatbot with the WhatsApp platform via its public Web API (eFigure 1). Participants' messages are routed through WhatsApp's instant messaging server to a separately constructed chatbot system, comprising both an administrative interface and the chatbot itself. Upon processing, the chatbot's response is returned to the WhatsApp server and immediately visible to the user. This process is virtually instantaneous, delivering a seamless user experience.

The chatbot system comprises three primary modules: i) Dialogue management module: This module logs all interactions between users and the chatbot, capturing essential details about users' activity and previous exchanges. The system's NLP component interprets each message's content, forwarding it to trigger relevant actions based on predefined rules. For example, if a message includes a specific keyword, the chatbot automatically replies with the corresponding information. Additionally, the module can initiate new conversations according to the intervention schedule; ii) User management module: This module tracks user engagement by recording conversation details. Administrators can associate users' WhatsApp numbers with the chatbot, monitor intervention progress, and identify any disconnections; and iii) Multimedia management module: Supporting media functionality, this module enables the chatbot to handle image and video exchanges, allowing users to upload, send, and receive multimedia content as part of their interactions.

2) A co-creation approach to develop the chatbot workflow and key question-answer (QA) database

First, in-depth interviews of five MSM and two experienced HIV testing administrators were first conducted to understand concerns and frequently asked questions (FAQ) related to HIVST and HIV testing, and users' expectation of the chatbot. Fieldnotes were taken immediately after each interview. The interviews were transcribed for thematic analysis, and the themes generated were discussed in a panel consisting of investigators (experts in public health, HIV prevention, health communication, and health psychology), CBO staff, and five local MSM, in order to enrich the findings and ensure their trustworthiness. The panel held multiple meetings, taking the findings in our groundwork and in-depth interviews into account for preparing the chatbot workflow and comprehensive question-answer (QA) pairs related to HIV, HIVST and HIV testing. Five other MSM were invited to review the chatbot workflow and QA pairs. Participants were invited to share their opinions on important questions that were missed, whether the answers were useful and appropriate to address their concerns, and any practical suggestion to modify the answers and chatbot workflow. In addition, five CBO staff were invited to review the chatbot workflow and provide feedback. The discussion with MSM and CBO staff was audio-recorded with participants' consent. Two independent researchers listened to the tapes and drew out key suggestions, which were then discussed in panel meeting to finalize the key QA database and chatbot workflow.

3) Conversation mechanism

Our chatbot interacted with users based on pre-defined rules (e.g., retrieved pre-specified responses in the key QA database), and could not generate responses beyond the rules. The design was conservative, but was able to reduce hallucination issues.[8] The chatbot uses NLP functions to interpret and understand users' input/questions, and retrieves most relevant responses from the key QA database using keyword matching. If the chatbot cannot match the user's input with a relevant response, it replies, "I am not confident about an answer to your question." After three consecutive off-topic inputs, the chatbot suggests a new conversation topic. When users request additional

© 2025 Chen S et al. *JAMA Network Open.*

information on a topic, the chatbot provides varied responses within the key QA database to add depth to the topic, and it can repeat responses upon request. If a conversation is interrupted, the chatbot cancels any incomplete inputs and invites the user to re-enter their message to ensure clarity and continuity. The chatbot was trained to answer questions based on the key QA database as accurately as possible until convergence was reached.

4) Pilot testing of HIVST-Chatbot

A total of 30 MSM who were HIV-negative or unknown sero-status were invited to use the HIVST-Chatbot. With informed consent, users' interactions with the chatbot were retrieved and reviewed by the research team. The chatbot ran smoothly in the pilot testing, and all users were satisfied with its performance and interface. The research team did not observe erroneous recommendation/responses made by the chatbot.

5) Data safety

Participants' chat history with the HIVST-Chatbot was protected by the confidential agreement with WhatsApp. The data was encrypted on the Chatbot server, and would not be used other parties. The chat history was removed from the server once the project is completed. Until then, the participants' chat history with the HIVST-Chatbot was stored in the Chatbot server and protected by passwords, with only the principal investigator having access to the data. With these measures, we believed the risk of a data breach should be minimal.

eFigure 1. Architecture of the Chatbot system

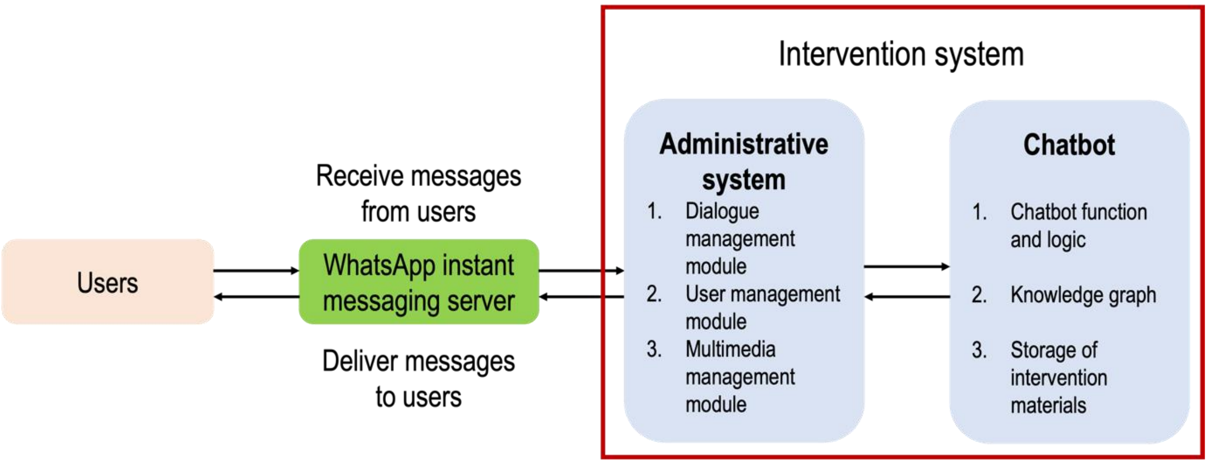

This figure shows the architecture of the chatbot system by adopting the WhatsApp platform to implement the Chatbot. The Chatbot is integrated with WhatsApp through its public Web API services. Participants' messages are sent to WhatsApp's instant messaging server and a separately constructed Chatbot system (an administrative system and the Chatbot). The Chatbot system processes and sends a message back to the WhatsApp instant messaging server. Finally, the users can view the message sent by the Chatbot. The entire process spans less than a second without a sluggish feel for users

eTable 1. Comparing the baseline characteristics between participants who completed Month 6 follow-up evaluation and those who were lost to follow up

	HIVST-chatbot (n=266)			HIVST-OIC (n=265)		
	Being followed up (n=240)	Loss-to-follow-up (n=26)	Two-sided p values	Being followed up (n=230)	Loss-to-follow-up (n=35)	Two-sided p values
	n (%)	n (%)		n (%)	n (%)	
**Sociodemographic characteristics**						
Age group, years						
18-24	24 (10.0)	3 (11.5)		29 (12.6)	6 (17.1)	
25-34	101 (42.1)	16 (61.6)		104 (45.2)	19 (54.3)	
35-44	72 (30.0)	5 (19.2)		61 (26.5)	7 (20.0)	
≥45	43 (17.9)	2 (7.7)	0.22	36 (15.7)	3 (8.6)	0.47
Relationship status						
Currently single	195 (81.3)	21 (80.8)		191 (83.0)	28 (80.0)	
Cohabited or married with a man	42 (17.5)	5 (19.2)		38 (16.5)	7 (20.0)	
Cohabited or married with a woman	3 (1.2)	0 (0.0)	0.83	1 (0.5)	0 (0.0)	0.82
Education level						
Secondary or below	42 (17.5)	3 (11.5)		32 (13.9)	8 (22.9)	
Tertiary and above	198 (82.5)	23 (88.5)	0.44	198 (86.1)	27 (77.1)	0.17
Current employment status						
Full-time	187 (77.9)	19 (73.1)		178 (77.4)	28 (80.0)	
Part-time/self-employed/unemployed/ retired/students	53 (22.1)	7 (26.9)	0.58	52 (22.6)	7 (20.0)	0.73
Monthly personal income, HK$ (US$)						
<20,000 (2,564)	71 (29.6)	9 (34.6)		72 (31.3)	10 (28.6)	
≥20,000 (2,564)	168 (70.0)	17 (65.4)		157 (68.3)	25 (71.4)	

© 2025 Chen S et al. *JAMA Network Open.*

Refuse to disclose	1 (0.4)	0 (0.0)	0.83	1 (0.4)	0 (0.0)	0.87
Sexual orientation						
Gay	217 (90.4)	22 (84.6)		209 (90.9)	30 (85.7)	
Bisexual	22 (9.2)	4 (15.4)		19 (8.3)	4 (11.4)	
Heterosexual	1 (0.4)	0 (0.0)		1 (0.4)	0 (0.0)	
Uncertain	0 (0.0)	0 (0.0)	0.57	1 (0.4)	1 (2.9)	0.40
**Health conditions**						
History of COVID-19						
No	47 (19.6)	5 (19.2)		40 (17.4)	7 (20.0)	
Yes	193 (80.4)	21 (80.8)	0.97	190 (82.6)	28 (80.0)	0.71
COVID-19 vaccination doses						
0-1	12 (5.0)	3 (11.5)		8 (3.5)	2 (5.7)	
2	25 (10.4)	3 (11.5)		34 (14.8)	9 (25.7)	
≥3	203 (84.6)	20 (77.0)	0.37	188 (81.7)	24 (68.6)	0.19
STI history						
No	176 (73.3)	19 (73.1)		169 (73.5)	28 (80.0)	
Yes	64 (26.7)	7 (26.9)	0.98	61 (26.5)	7 (20.0)	0.41
**HIV testing in the past six months**						
HIV testing						
No	122 (50.8)	18 (69.2)		118 (51.3)	24 (68.6)	
Yes	118 (49.2)	8 (30.8)	0.07	112 (48.7)	11 (31.4)	0.06
Use of specific type of HIV testing, yes						
HIV testing at community-based organizations in Hong Kong	74 (30.8)	3 (11.5)	0.04	72 (31.3)	5 (14.3)	0.04
HIV testing at governmental clinics in Hong Kong	16 (6.7)	2 (7.7)	0.84	24 (10.4)	4 (11.4)	0.86
HIV testing at private clinics in Hong Kong	4 (1.7)	1 (3.8)	0.44	2 (0.9)	0 (0.0)	0.58
HIV testing at other organizations in Hong Kong	0 (0.0)	0 (0.0)	N.A.	1 (0.4)	1 (2.9)	0.12
HIV testing in places other than Hong Kong	3 (1.3)	0 (0.0)	0.57	5 (2.2)	0 (0.0)	0.38

HIV self-testing	43 (17.9)	3 (11.5)	0.41	33 (14.3)	4 (11.4)	0.64
**Sexual behaviors in the past six months**						
Anal intercourse with regular male sex partner						
No	62 (25.8)	10 (38.5)		56 (24.3)	8 (22.9)	
Yes	178 (74.2)	16 (61.5)	0.17	174 (75.7)	27 (77.1)	0.85
Anal intercourse with non-regular male sex partner						
No	129 (53.8)	16 (61.5)		122 (53.0)	20 (57.1)	
Yes	111 (46.3)	10 (38.5)	0.45	108 (47.0)	15 (42.9)	0.65
Anal intercourse with male sex worker						
No	234 (97.5)	25 (96.2)		219 (95.2)	34 (97.1)	
Yes	6 (2.5)	1 (3.8)	0.68	11 (4.8)	1 (2.9)	0.61
Condomless anal intercourse with men						
No	111 (46.3)	13 (50.0)		98 (42.6)	12 (34.3)	
Yes	129 (53.7)	13 (50.0)	0.72	132 (57.4)	23 (65.7)	0.35
Multiple male sex partnerships						
No	121 (50.4)	15 (57.7)		109 (47.4)	19 (54.3)	
Yes	119 (49.6)	11 (42.3)	0.48	121 (52.6)	16 (45.7)	0.45
Sexualized drug use						
No	220 (91.7)	23 (88.5)		212 (92.2)	30 (85.7)	
Yes	20 (8.3)	3 (11.5)	0.58	18 (7.8)	5 (14.3)	0.21
**Other HIV/STI prevention service utilization in the past six months**						
STI testing						
No	147 (61.3)	19 (73.1)		136 (59.1)	25 (71.4)	
Yes	93 (38.7)	7 (26.9)	0.24	94 (40.9)	10 (28.6)	0.17
PrEP use						
No	208 (86.7)	24 (92.3)		194 (84.3)	31 (88.6)	

© 2025 Chen S et al. *JAMA Network Open.*

Yes	32 (13.3)	2 (7.7)	0.41	36 (15.7)	4 (11.4)	0.52
Use of other HIV/STI prevention services (e.g., receiving condoms, peer education, webinar/seminar/workshops)						
No	139 (57.9)	19 (73.1)		129 (56.1)	22 (62.9)	
Yes	101 (42.1)	7 (26.9)	0.14	101 (43.9)	13 (37.1)	0.45

STI: sexually transmitted infections

PrEP: pre-exposure prophylaxis

© 2025 Chen S et al. *JAMA Network Open.*

eTable 2. Comparisons of primary outcomes among different sub-groups of participants between HIVST-Chatbot and HIVST-OIC groups

Sub-groups	HIVST-chatbot n/N (%)	HIVST-OIC n/N (%)	Proportion difference (95%CI)	RR (95%CI)	ARR (95%CI)	NNT (95%CI)	One-sided p values
**HIVST uptake in different sub-groups**							
CAI with men at baseline							
No	90/124 (72.6)	80/110 (72.7)	-0.2% (-9.8%, 9.5%)	1.00 (0.87, 1.14)	0.00 (-0.10, 0.09)	-682 (-10.26, 10.57)	0.50
Yes	105/142 (73.9)	117/155 (75.5)	-1.5% (-9.9%, 6.8%)	0.98 (0.88, 1.09)	-0.02 (-0.10, 0.07)	-64.93 (-10.15, 14.78)	0.43
Multiple male sex partnerships at baseline							
No	99/136 (72.8)	93/128 (72.7)	0.1% (-8.9%, 9.2%)	1.00 (0.88, 1.13)	0.00 (-0.09, 0.09)	725.33 (-11.26, 10.92)	0.50
Yes	96/130 (73.8)	104/137 (75.9)	-2.1% (-10.8%, 6.7%)	0.97 (0.87, 1.09)	-0.02 (-0.11, 0.07)	-48.40 (-9.26, 14.99)	0.40
HIV testing at baseline							
No	96/140 (68.6)	96/142 (67.6)	1.0% (-8.2%, 10.1%)	1.01 (0.89, 1.16)	0.01 (-0.08, 0.10)	103.54 (-12.25, 9.90)	0.51
Yes	99/126 (78.6)	101/123 (82.1)	-3.5% (-11.8%, 4.7%)	0.96 (0.86, 1.06)	-0.04 (-0.12, 0.05)	-28.23 (-8.46, 21.13)	0.29
**Counseling support in different sub-groups**							

© 2025 Chen S et al. *JAMA Network Open.*

CAI with men at baseline							
No	81/90 (90.0)	48/80 (60.0)	30.0% (19.6%, 40.4%)	1.50 (1.28, 1.76)	0.30 (0.20, 0.40)	3.33 (2.48, 5.10)	<0.001
Yes	100/105 (95.2)	72/117 (61.5)	33.7% (25.6%, 41.9%)	1.55 (1.37, 1.75)	0.34 (0.26, 0.42)	2.97 (2.39, 3.91)	<0.001
Multiple male sex partnerships at baseline							
No	92/99 (92.9)	62/93 (66.7)	26.3% (17.2%, 35.4%)	1.39 (1.23, 1.59)	0.26 (0.17, 0.35)	3.81 (2.83, 5.82)	<0.001
Yes	89/96 (92.7)	58/104 (55.8)	36.9% (27.8%, 46.1%)	1.66 (1.43, 1.93)	0.37 (0.28, 0.46)	2.71 (2.17, 3.60)	<0.001
HIV testing at baseline							
No	89/96 (92.7)	56/96 (58.3)	34.4% (25.0%, 47.4%)	1.59 (1.37, 1.90)	0.34 (0.25, 0.44)	2.91 (2.29, 4.00)	<0.001
Yes	92/99 (92.9)	64/101 (63.4)	29.6% (20.6%, 38.5%)	1.47 (1.28, 1.67)	0.30 (0.21, 0.39)	3.38 (2.60, 4.85)	<0.001

HIVST: HIV self-testing
RR: relative risk
ARR: absolute risk reduction
NNT: number needed to treat
CI: confidence interval

© 2025 Chen S et al. *JAMA Network Open.*

eTable 3. Within-group comparisons of primary and secondary outcomes among HIVST-Chatbot and HIVST-OIC groups

	HIVST-Chatbot n/N (%)	HIVST-OIC n/N (%)
Total participants	266	265
**Primary outcomes**		
Use of any HIVST		
Baseline	46/266 (17.3)	37/265 (14.0)
Month 6 [a]	216/266 (81.2)	227/265 (85.7)
Month 6 versus Baseline (two-sided p values)	<0.001	<0.001
Any types of counseling support alongside with HIVST (among all HIVST users)		
Baseline	12/216 (5.6)	19/227 (8.8)
Month 6 [a]	197/216 (91.2)	142/227 (62.6)
Month 6 versus Baseline (two-sided p values)	<0.001	<0.001
**Secondary outcomes**		
CAI with men		
Baseline	142/266 (53.4)	155/265 (58.5)
Month 6 [a]	124/266 (46.6)	144/265 (54.3)
Month 6 versus Baseline (two-sided p values)	0.054	0.32
Multiple male sex partnerships		
Baseline	130/266 (48.9)	137/265 (51.7)
Month 6 [a]	120/266 (45.1)	131/265 (49.4)
Month 6 versus Baseline (two-sided p values)	0.34	0.37
Uptake of HIV testing other than HIVST		
Baseline	92/266 (34.6)	100/265 (37.7)
Month 6 [a]	54/266 (20.3)	70/265 (26.4)

© 2025 Chen S et al. *JAMA Network Open.*

Month 6 versus Baseline (two-sided p values)	<0.001	0.004
Uptake of any type of HIV testing		
Baseline	126/266 (47.4)	123/265 (46.4)
Month 6 [a]	228/266 (85.7)	238/265 (89.8)
Month 6 versus Baseline (two-sided p values)	<0.001	<0.001

HIVST: HIV self-testing
CI: confidence interval
CAI: condomless anal intercourse
[a] Multiple imputation was performed to replace the missing values at Month 6. The Markov chain Monte Carlo method was used to impute the missing values of outcomes at Month 6 separately in a randomized group

© 2025 Chen S et al. *JAMA Network Open.*

eTable 4. Process evaluation among participants who completed the process evaluation at Month 6

Domains	Items	HIVST-chatbot n/N (%)	HIVST-OIC n/N (%)	Two-sided p values
Compared to facility-based HIV testing	Convenience	137/153 (89.5%)	138/174 (79.3%)	0.01
	Protects privacy	136/153 (88.9%)	145/174 (83.3%)	0.15
	Reduces discrimination	117/153 (76.4%)	120/174 (68.9%)	0.13
	Provides support	82/153 (53.6%)	105/174 (60.3%)	0.22
	Helpful to perform HIVST	89/153 (58.2%)	102/174 (58.6%)	0.93
Perceptions of HIVST-chatbot	Less affected by time/location	147/153 (96.1%)	N.A.	N.A.
	Similar to facility testing	96/153 (62.7%)	N.A.	N.A.
Behavioral engagement (TWEETS)	Part of daily routine	96/153 (62.7%)	119/174 (68.4%)	0.28
	Easy to use	135/153 (88.2%)	152/174 (87.4%)	0.81
	Information available as needed	130/153 (85.0%)	145/174 (83.3%)	0.69
	Behavior Engagement Sub-scale, mean (SD) [a]	12.2 (2.2)	12.4 (1.9)	0.38
Cognitive engagement (TWEETS)	Addresses barriers in HIVST	119/153 (77.8%)	140/174 (80.5%)	0.08
	Motivates HIVST	120/153 (78.4%)	133/174 (76.4%)	0.67
	Improves insight in HIVST	117/153 (76.4%)	146/174 (83.9%)	0.09
	Cognitive Engagement Sub-scale, mean (SD) [b]	12.2 (2.3)	12.6 (2.1)	0.10
Affective engagement	Enjoy using service	114/153 (74.5%)	126/174 (72.4%)	0.67

© 2025 Chen S et al. *JAMA Network Open.*

(TWEETS)	Enjoy seeing progress	86/153 (56.2%)	90/174 (51.7%)	0.42
	Service fits me	126/153 (82.4%)	141/174 (81.0%)	0.76
	Affective Engagement Sub-scale, mean (SD) [c]	11.8 (2.4)	11.7 (2.0)	0.68
Intention & Recommendation	Intend to use in 6 months	119/153 (77.8%)	145/174 (83.3%)	0.21
	Recommend to friends	120/153 (78.4%)	151/174 (86.8%)	0.05

HIVST: HIV self-testing
TWEETS: Twente Engagement with eHealth Technologies Scale
[a] Behavior Engagement Sub-scale, 3 items, scale score: 3-15, a higher score indicated more behavior engagement with HIVST-chatbot or HIVST-OIC; Cronbach's alpha: 0.79 and 0.70 in HIVST-chatbot and HIVST-OIC groups respectively; one factor was identified by exploratory factor analysis, explaining for 71.3% and 65.0 % of the total variance in HIVST-chatbot and HIVST-OIC groups respectively.
[b] Cognitive Engagement Sub-scale, 3 items, scale score: 3-15, a higher score indicated more cognitive engagement with HIVST-chatbot or HIVST-OIC; Cronbach's alpha: 0.77 and 0.79 in HIVST-chatbot and HIVST-OIC groups respectively; one factor was identified by exploratory factor analysis, explaining for 68.8% and 71.0 % of the total variance in HIVST-chatbot and HIVST-OIC groups respectively.
[c] Affective Engagement Sub-scale, 3 items, scale score: 3-15, a higher score indicated more affective engagement with HIVST-chatbot or HIVST-OIC; Cronbach's alpha: 0.82 and 0.72 in HIVST-chatbot and HIVST-OIC groups respectively; one factor was identified by exploratory factor analysis, explaining for 73.5% and 64.3 % of the total variance in HIVST-chatbot and HIVST-OIC groups respectively.

© 2025 Chen S et al. *JAMA Network Open.*

eTable 5. Economic evaluation of the HIVST-OIC compared with the HIVST-Chatbot among participants

Outcomes	Cost (US$)	Effectiveness	Per person cost (US$)	ICER
**Outcome 1: Number of people receiving HIVST**				
HIVST-OIC	30,885.2	197	156.8	
HIVST-Chatbot	27,258.8	195	139.8	1813.2
**Outcome 2: Number of HIVST users receiving any counseling support**				
HIVST-OIC	30,885.2	120	257.4	
HIVST-Chatbot	27,258.8	181	150.6	Dominated

HIVST: HIV self-testing
ICER: Incremental cost-effectiveness ratios
Dominated: The HIVST-Chatbot group is both more effective and less costly than the HIVST-OIC.
US$ United States dollar

© 2025 Chen S et al. *JAMA Network Open.*

eTable 6. Costs items over a 6-month time horizon

Variable costs	HIVST-chatbot group costs (US$)	HIVST-OIC group costs (US$)
Packaging	672	644
Instruction booklet	240	230
HIV self-test kits	9141.9	8747.5
Blood taking needle	0.08	0.16
Alcohol pad	0.24	0.47
Health promotional video design	4294.87	4294.87
Chatbot development and maintenance [1]	9871.79	N.A.
Simplified counseling costs	N.A.	10393.77
Comprehensive counseling costs	N.A.	962.39
Incentives	1531.2	1467.4
Delivery cost of HIV self-test kits	768	736
Laboratory confirmation for HIV	371.8	557.7
Staff time (testing arrangements for counseling and liaison with collaborating organization)	N.A.	2301.28
**Fixed costs**		
Internet	153.6	153.6
Utilities (electricity)	38.4	38.4
Telephone bill	N.A.	10.3
Computers [2]	133.8	267
Desks [3]	26.7	53.34
Desk chairs [3]	14.4	27
Total **(US$)**	27,258.8	30,885.2

[1] The cost of a chatbot development and maintenance was annualized over a five-year period at a discount rate of 3%, and the 6-month pro-rata costs valued here
[2] The cost of a computer was annualized over a seven-year period at a discount rate of 3%, and the 6-month pro-rata costs valued here.
[3] The cost of desk and chairs was annualized over a five-year period at a discount rate of 3% and the 6-month pro-rata costs valued here.

© 2025 Chen S et al. *JAMA Network Open.*

References:

1. Wang Z, Lau JTF, Ip M, et al. A Randomized Controlled Trial Evaluating Efficacy of Promoting a Home-Based HIV Self-Testing with Online Counseling on Increasing HIV Testing Among Men Who Have Sex with Men. *AIDS Behav*. Jan 2018;22(1):190-201. doi:10.1007/s10461-017-1887-2
2. Kwan N, Wong A, Fang Y, Wang Z. 'Get an early check - Chrysanthemum tea': An outcome evaluation of a multimedia campaign promoting HIV testing among men who have sex with men in Hong Kong. *HIV Med*. May 2018;19(5):347-354. doi:10.1111/hiv.12583
3. Tang W, Wei C, Cao B, et al. Crowdsourcing to expand HIV testing among men who have sex with men in China: A closed cohort stepped wedge cluster randomized controlled trial. *PLoS Med*. Aug 2018;15(8):e1002645. doi:10.1371/journal.pmed.1002645
4. Zhong F, Tang W, Cheng W, et al. Acceptability and feasibility of a social entrepreneurship testing model to promote HIV self-testing and linkage to care among men who have sex with men. *HIV Med*. May 2017;18(5):376-382. doi:10.1111/hiv.12437
5. Wang X, Tang Z, Wu Z, Nong Q, Li Y. Promoting oral HIV self-testing via the internet among men who have sex with men in China: a feasibility assessment. *HIV Med*. May 2020;21(5):322-333. doi:10.1111/hiv.12830
6. Pong S, Fowler RA, Mitsakakis N, et al. Noninferiority Margin Size and Acceptance of Trial Results: Contingent Valuation Survey of Clinician Preferences for Noninferior Mortality. *Med Decis Making*. Aug 2022;42(6):832-836. doi:10.1177/0272989x221099493
7. Tweed CD, Quartagno M, Clements MN, et al. Exploring different objectives in non-inferiority trials. *Bmj*. Jun 17 2024;385:e078000. doi:10.1136/bmj-2023-078000
8. Lavrinovics E, Biswas R, Bjerva J, Hose K. Knowledge Graphs, Large Language Models, and Hallucinations: An NLP Perspective. *Journal of Web Semantics*. 2025/05/01/ 2025;85:100844. doi:https://doi.org/10.1016/j.websem.2024.100844

© 2025 Chen S et al. *JAMA Network Open.*
