# Supplementary material for: Chatbot-Delivered Real-Time Support to Improve HIV Self-Testing Rates: A Randomized Clinical Trial
Source: JAMA Netw Open. 2025 Nov 24;8(11):e2544821. doi: 10.1001/jamanetworkopen.2025.44821 (PMC12645333; doi:10.1001/jamanetworkopen.2025.44821)
Supplement: Supplement 3. — Data Sharing Statement [file jamanetwopen-e2544821-s003.pdf]

Data Sharing Statement

Chen. Chatbot-Delivered Real-Time Support to Improve HIV Self-Testing Rates. *JAMA Netw Open*. Published November 24, 2025. doi:10.1001/jamanetworkopen.2025.44821

Data
Additional Information: ClinicalTrial.gov identifier: NCT05796622 (https://clinicaltrials.gov/study/NCT05796622?cond=NCT05796622&rank=1)
Data available: No

Additional Information
Explanation for why data not available: The datasets generated and/or analyzed during the current study are not publicly available as they contain sensitive personal behaviors but are available from the corresponding author on reasonable request.
